# Transcriptomic Complexity of *Aspergillus terreus* Velvet Gene Family under the Influence of Butyrolactone I

**DOI:** 10.3390/microorganisms5010012

**Published:** 2017-03-14

**Authors:** Elina K. Palonen, Sheetal Raina, Annika Brandt, Jussi Meriluoto, Tajalli Keshavarz, Juhani T. Soini

**Affiliations:** 1 Biochemistry, Faculty of Science and Engineering, Åbo Akademi University, Åbo FI-20520, Finland; annika.brandt@abo.fi (A.B.); jussi.meriluoto@abo.fi (J.M.); 2 Department of Life Sciences, University of Westminster, London W1W 6UW, UK; rainas2@googlemail.com (S.R.); t.keshavarz@westminster.ac.uk (T.K.); 3 Faculty of Life Sciences and Business, Turku University of Applied Sciences, Åbo FI-20520, Finland; juhani.soini@turkuamk.fi

**Keywords:** *Aspergillus terreus*, filamentous fungi, velvet, LaeA, secondary metabolism, conidiation, quorum sensing, butyrolactone I, transcriptome sequencing, gene expression

## Abstract

Filamentous fungi of the *Ascomycota* phylum are known to contain a family of conserved conidiation regulating proteins with distinctive velvet domains. In Aspergilli, this velvet family includes four proteins, VeA, VelB, VelC and VosA, and is involved in conidiation and secondary metabolism along with a global regulator LaeA. In *A. terreus*, the overexpression of LaeA has been observed to increase the biogenesis of the pharmaceutically-important secondary metabolite, lovastatin, while the role of the velvet family has not been studied. The secondary metabolism and conidiation of *A. terreus* have also been observed to be increased by butyrolactone I in a quorum-sensing manner. An enlightenment of the interplay of these regulators will give potential advancement to the industrial use of this fungus, as well as in resolving the pathogenic features. In this study, the *Aspergillus terreus* MUCL 38669 transcriptome was strand-specifically sequenced to enable an in-depth gene expression analysis to further investigate the transcriptional role of butyrolactone I in these processes. The sequenced transcriptome revealed intriguing properties of the velvet family transcripts, including the regulator *laeA*, and uncovered the *velC* gene in *A. terreus*. The reliability refining microarray gene expression analysis disclosed a positive regulatory role for butyrolactone I in *laeA* expression, as well as an influence on the expression of the canonical conidiation-regulating genes under submerged culture. All of this supports the suggested regulative role of butyrolactone I in *A. terreus* secondary metabolism, as well as conidiation.

## 1. Introduction

The filamentous fungus *Aspergillus terreus* is commonly isolated from soil rhizospheres [1,2], as well as from decaying organic matter [3] and has been reported to produce a plenitude of secondary metabolites, such as itaconic acid, putative lipases and cellulases [3,4,5,6], with potential industrial importance, lovastatin with medical significance as a serum cholesterol lowering agent [7] and toxins citrinin, emodin and gliotoxin [8,9,10,11], as well as other compounds, including aspulvinones, asterriquinones, butyrolactones and (+)-geodin [12,13,14,15,16,17]. The exogenous addition of butyrolactone I (methyl 4-hydroxy-2-[[4-hydroxy-3-(3-methylbut-2-enyl)phenyl]methyl]-3-(4-hydroxyphenyl)-5- oxofuran-2-carboxylate) has been observed to enhance hyphal branching and sporulation, increase the produced amount of secondary metabolites lovastatin, sulochrin and itself and was thus suggested to play a quorum-sensing role in this fungus, i.e., implicating autoregulatory cell-to-cell communication in order to adapt to the environmental conditions [18,19,20]. In addition to the pharmaceutically- and industrially-important increase in secondary metabolism regarding lovastatin, the observed effect on morphological development is of significance, as well. *A. terreus* has been observed amongst the fungal pathogens *Aspergillus fumigatus*, *Aspergillus flavus* and *Aspergillus niger* to cause invasive aspergillosis in immunocompromised patients. *A. terreus* has also shown resistance to amphotericin B, complicating aspergillosis therapy [21]. Sporulation may play an important role in the pathogenicity by increasing the defence against diverse environmental conditions in filamentous fungi. Recently, *A. terreus* spores were suggested to have an important, but different role than the spores of *A. fumigatus* in the invasive aspergillosis by remaining vital in the macrophages after phagocytosis even in immunocompetent hosts [22].

**Figure 1:**
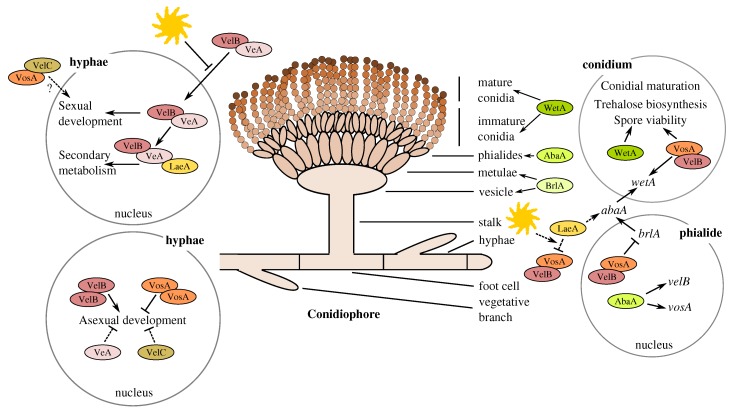
A schematic description of the cellular differentiation of the model fungus *A. nidulans* with the suggested role of the velvet gene family. Modified and based on Park and Yu (2012) and Sarikaya Bayram et al. (2010) [32,34].

The developmental growth phases leading to sexual or asexual sporulation have been most thoroughly studied in the model fungus *Aspergillus nidulans*. Asexual sporulation and conidiation of Aspergilli begin when the vegetative growth ceases due to various changes in the growth conditions, such as intensity of light, nutrition and other environmental stress factors. In static, aerial growth conditions, the first morphological phase of conidiation is hyphal branching, specifically stalk emergence and growth on specific foot cells. When optimum length is achieved, the stalk elongation ceases, and the formation of a vesicle at the tip of the stalk takes place, which is followed by budding of numerous metulae. The next step is the formation of phialides on the metulae, followed by the production of numerous conidia and their maturation process [23]. The three core regulator genes, *brlA*, *abaA* and *wetA*, are sequentially involved in the control of this cellular differentiation in *A. nidulans*. The *brlA* gene regulates the proceeding from the stalk to vesicle and metulae formation; *abaA* controls the phialide stage; while *wetA* is required for the conidia maturation process [24,25,26,27,28,29]. In addition, a trehalose biosynthesis conducting gene, *tpsA*, is observed to be involved in the conidia maturation, as well (Figure 1) [30,31,32,33].

The connection between sporulation and secondary metabolism in *Ascomycetes* has been observed to include a specific velvet gene family with a regulative role. The *Aspergillus* species have been reported to have four different velvet family proteins, VeA, VelB, VelC and VosA, that contain the canonical velvet domains and a global regulator protein LaeA containing a methyltransferase domain. The VeA component of the velvet family was the first to be identified and further studied in *A. nidulans*, revealing its role in the control of sporulation and secondary metabolism [35,36]. The velvet family members were thereafter discovered to form multi-subunit complexes with diverse homodimeric, heterodimeric and trimeric variants depending on the cellular growth phase and cell compartment leading to differential control of development, conidiation and secondary metabolism. The factors affecting the composition of the different complexes have been observed to correspond to the conditions, including light or dark environment, which regulating the cellular differentiation from hyphae to either asexual or sexual development. The velvet family has most thoroughly been studied in the model fungus *A. nidulans* (Figure 1) [31,32,33,37,38], while in *A. terreus*, the function of LaeA has been only briefly in focus [39]. The VelB-VeA-LaeA complex was discovered to distinctly coordinate secondary metabolism along with the conidia and cleistothecia formation balance in *A. nidulans* [37].

In *A. terreus*, sporulation has been observed to be enhanced under the influence of exogenous butyrolactone I along with secondary metabolism [18]. In this study, we aim to further illuminate the effect of exogenous addition of butyrolactone I on transcriptional level. The transcriptome of *A. terreus* strain MUCL 38669 was sequenced to discover and clarify transcriptional differences between the annotated strain NIH2624 and strain MUCL 38669, which was used in our previous studies [19,20]. In this study, we discovered butyrolactone I to upregulate *laeA* gene expression, confirming the suggested role for butyrolactone I in secondary metabolism. We describe also the accumulation levels and alternative splicing of *laeA* and the velvet family transcripts *veA*, *velB*, *vosA*, as well as *velC*, whose presence in *A. terreus* strain MUCL 38669 and genomic locus in *A. terreus* strain NIH2624 was uncovered by the strand-specific whole transcriptome sequencing under the increased butyrolactone I biogenesis.

## 2. Materials and Methods

### 2.1. Strain and Chemicals

All culture materials, including the *Aspergillus terreus* strain MUCL 38669, used in this study are the same as was used in our previous study [19] and are described in Supplementary Methods (File S1).

### 2.2. Culture Conditions

*A. terreus* MUCL 38669 was cultured under shaken, submerged growth conditions in three biological replicates for nine days. The growth conditions were the same as in our previous studies of secondary metabolism [19,20]. Briefly, *A. terreus* MUCL 38669 spores were maintained on yeast and malt extract (YME) agar slants. Collected spores (final concentration $10^{7}$/mL) were incubated in 100 mL of inoculation medium for 25 h at 27 °C. One hundred millilitres of glucose, peptonised milk, yeast extract and lactose containing (GPY-L) production medium (pH 7.4) were inoculated with 10 mL of the inoculation medium and incubated at 27 °C for 216 h. More detailed description of the used growth conditions are described in the Supplementary Methods (File S1).

### 2.3. Addition of Butyrolactone I

Exogenous butyrolactone I was added at 24 h, 96 h and 120 h post-inoculation (Test Sets 1, 2 and 3, respectively), and each of the test sets, as well as the control set (no butyrolactone I added) were sampled at 24 h, 48 h, 96 h, 120 h, 144 h and 216 h post-inoculation. The exogenous butyrolactone I was dissolved in ethanol and added to the test cultures to a final concentration of 100 nM.

### 2.4. RNA Extraction

The total RNA used in this study was derived from our previous study [19] (see File S1 for details) and had been extracted using the Qiagen RNeasy Plant Mini Kit (Qiagen, Sollentuna, Sweden) from the snap-frozen (−80 °C) *A. terreus* mycelia sampled at the six time points and was stored at −80 °C.

### 2.5. Gene Expression Analysis Using Microarrays

The microarray raw data, i.e., the extracted one-colour signal intensities of the microarray images, were obtained from our previous study [19] using the custom Agilent 4 × 44 K format (Agilent Technologies Inc., Wilmington, DE, USA) to be further analysed. The available genomic sequence information of *A. terreus* strain NIH2624 (Broad Institute’s genome sequencing programme [40]) had been used as the platform for the microarray design, while the strain cultured in the study was MUCL 38669. The statistical analysis of the microarray results was performed using the same algorithms of the statistical computing language and environment R (R Development Core Team (2011) [41,42] version 2.14.1) and the linear models for the microarray data (limma) package [43,44,45] as in the previous study [19] (see File S1 for further details) with one exception regarding the 60-mer oligonucleotide microarray probes that had been designed based on the genomic sequence of strain NIH2624. The following bioinformatic series of steps was applied to exclude the unreliable probes from the further deep analysis. The probe sequences were aligned with the obtained transcriptome of *A. terreus* MUCL 38669 using Nucleotide-Nucleotide BLAST (Version 2.2.29+) [46,47]. A probe was considered reliable when the following conditions were fulfilled: (I) if the length of the alignment was 58 nt or less, no mismatches were allowed; (II) if the length of the alignment was 59 nt, only 1 mismatch was allowed; (III) if the length of the alignment was 60 nt, only 2 mismatches were allowed at a maximum. The further statistical methods to discover the statistically-significant differential gene expression resulting from the exogenous addition of butyrolactone I were the same as used in our previous study [19] (see File S1 for further details). The microarray experiment had been performed in three biological replicates and in four technical replicates at the probe level. Differential gene expression (|log${}_{2}$FC| > 0.5) was considered as statistically significant if adjusted *p* < 0.05. See File S1, Figures S2 and S3 for a more detailed description.

### 2.6. Strand-Specific Transcriptome Sequencing

The method used for the synthesis of strand-specific double-stranded cDNA (ds cDNA) from the total RNA was a modified combination of protocols presented by Marioni et al. (2008) [48], Parkhomchuk et al. (2009) [49], Levin et al. (2010) [50] and the standard Illumina mRNA preparation protocol (Preparing Samples for Sequencing of mRNA, Part No. 1004898 Rev. A, Illumina Inc., San Diego, CA, USA) due to the lack of strand-specificity in the contemporary standard mRNA sequencing protocols. Briefly, the total RNA to be sequenced had been extracted from all 6 sampling time points of Test Set 3 of the *A. terreus* MUCL 38669 mycelia, where butyrolactone I had been added at 120 h p.i., obtained from the submerged culture in our previous study [19]. This set of total RNA was pooled to acquire the required amount of strand-specific ds cDNA for the high throughput DNA sequencing. The total RNA was purified to obtain mRNA using oligo-dT magnetic beads followed by fragmentation into short mRNA sequences. Next, the first-strand cDNA was synthesised using reverse transcriptase with a high amount of random hexamer primers. The second strand cDNA was synthesised using DNA ligase, DNA polymerase I and RNase H with dNTPs that contained dUTPs instead of dTTPs. From this step forwards, the Illumina mRNA preparation protocol was used according to the instructions with one exception. The dU-containing second strand was digested with uracil-N-glycosylase just before the library amplification step to obtain strand-specific sequencing results. See the Supplementary File S1 for a detailed description of the used protocol. The strand-specific library was sequenced with Illumina GAII followed by base-calling and calculation of the quality values for every base of the paired-end reads with Illumina’s Pipeline Analysis software resulting in raw data in the fastq format.

### 2.7. The Assembly of Strand-Specific RNA Sequence Data

The obtained transcriptome sequence data were trimmed to remove the multiplex adapters, PCR primers and low quality parts of the reads using a FASTX-Toolkit (Version 0.0.13) [51], and the resulting read sequence quality was evaluated with the FastQC programme (File S1, Table S1) [52]. Next, the trimmed paired-end reads were de novo assembled using Trinity (Version trinityrnaseq_r2012-03-17) [53,54,55] with the default parameters, including “jaccard_clip” to prevent an erroneous fusion of overlapping transcripts of a compact fungal genome, such as *A. terreus*, and the “kmer_method” used was “meryl” to maintain the strand specificity of the obtained sequenced transcriptome data. In addition, a Genome-guided Trinity pipeline (Beta, Version trinityrnaseq_r2013-02-25) [54,55,56] was used to resolve the structures of the transcripts following the software’s instructions [56]. Briefly, (I) GSNAP [57,58] was used to align the trimmed paired-end reads to the genomic sequence of *A. terreus* strain NIH2624; (II) Trinity was used to assembly the aligned reads; (III) GMAP [58,59] was used to align the obtained aligned transcripts back to the genomic sequence of *A. terreus* NIH2624; and (IV) PASA pipeline (Version PASA2-r20130605) [60,61,62] was used to assemble the newly-obtained aligned transcripts. Next, to capture the transcripts with differing exon-intron structures and alternative splicing, a comprehensive transcriptome database was created using the PASA pipeline as follows: the transcripts obtained from de novo assembly and genome-guided assembly were concatenated and aligned to the genomic sequence of *A. terreus* NIH2624 using the PASA pipeline scripts “Launch_PASA_pipeline.pl” and “build_comprehensive_transcriptome.dbi” with the following parameters: BLAT (Version 35x1) [63,64] and GMAP as the aligning applications, stringent alignment overlap of 30, maximum intron length of 2000, minimum nucleotide identity of 95% and minimum alignment length of 30%. The assembled transcripts with potential structural differences, i.e., the transcripts that had not passed the applied criteria, were saved for further analysis, whereas the transcripts that had passed the applied criteria regarding the alignment to *A. terreus* NIH2624 genome were extracted and re-assembled using a Minimo assembler of AMOS Assembler pipeline (Version 3.1.0) [65,66] instead of the PASA pipeline in order to retain the sequence differences between the two *A. terreus* strains (NIH2624 and MUCL 38669). The used parameters in the re-assembly with the Minimo assembler were as follows: quality score for bases within the clear range (GOOD_QUAL) 30, quality score for bases outside the clear range (BAD_QUAL) 30, minimum contig overlap (MIN_LEN) 20, minimum contig overlap identity percentage (MIN_IDENT) 100, strand-specificity (STRAND_SPEC) 1, alignment wiggle value (ALN_WIGGLE) 15, to export results in ACE format (ACE_EXP) 1, to export results in FASTA format (FASTA_EXP) 1.

### 2.8. Further Analysis of the Transcriptome Sequence Data

The obtained de novo and genome-guided transcripts of *A. terreus* strain MUCL 38669 (all of these transcripts originate from the extracted and subsequently pooled RNA) were first aligned with the genome of strain NIH2624 using BLAT (Version 35x1) [63,64], and the resulting alignment indexes were re-formatted into General Feature Format (gff3) format in order to view the aligned transcripts using Integrative Genomics Viewer (IGV, Version IGV_2.3.26) [67,68,69]. The alignment of the sequence reads with the genomic sequence of strain NIH2624 that was performed with GSNAP [57,58] during the Genome-Guided Trinity pipeline [54,55,56] was also viewed with IGV [67,68,69] in order to visualise the sense and antisense read coverages of the obtained de novo and genome-guided transcripts. The resulting mapped reads were quantified and normalised using Cufflinks (Version 2.2.1) tools Cuffquant and Cuffnorm [70] resulting in the number of fragments per kilobase of exon per million reads mapped (FPKM of pooled samples). A median of the read coverage over the genome was also calculated with a window size of 25 bp using the default parameters of the igvtools application of IGV [67,68,69] in order to obtain approximate transcription levels and maximum count values for the sequenced transcripts (represented as counts values in the Results Section). The annotated transcripts of strain NIH2624 (obtained from Broad Institute’s *A. terreus* genome sequencing programme [40]) were also included in the visualisation. A GENSCAN Web Server at MIT [71,72,73] was used to further resolve the putative intron-exon structures indicated by the aligned transcript reads if the read coverage of the sequenced transcripts was too low. The transcripts of interest were translated to putative proteins using the ExPASy Translate tool of Swiss Institute of Bioinformatics [74,75] with standard genetic code. The putative domains of the translated transcripts of interest were searched utilising the Integrated Resource of Protein Families, Domains and Functional Sites Database of UniProt Consortium (InterPro [76,77,78]) and NCBI Conserved domains database ([79,80]). An NCBI protein BLAST program [81] was used in order to find the putative homologues for the translated transcripts of interest. To build the phylogenetic trees, a web server, Phylogeny.fr [82,83], was used with the following programmes. Alignment was performed with MUSCLE (default settings), curated by Gblocks (default settings), and the trees were reconstructed using PhyML with the maximum likelihood method with the bootstrapping to estimate the internal branch reliability (default settings).

### 2.9. Accession Numbers

The following NCBI GenBank database accession numbers were obtained regarding the *A. terreus* MUCL 38669 transcripts studied in this study: KX470762 (*abaA*), KX470763 (*wetA*), KX470770 (*tpsA*), KY425747 (*laeA* transcript variant 1; LaeA-*α*), KY425748 (*laeA* transcript variant 2; LaeA-*α*), KY425749 (*laeA* transcript variant 3; LaeA-*β*), KY425750 (*laeA* transcript variant 4; LaeA-*β*), KY425751 (*vosA* transcript variant 1; VosA-*α*), KY425752 (*vosA* transcript variant 2; VosA-*β*), KY425753 (*vosA* transcript variant 3; VosA-*γ*), KY425754 (*vosA* transcript variant 4; VosA-*δ*), KY425755 (*vosA* transcript variant 5; VosA-*ϵ*), KY425756 (*vosA* transcript variant 6; VosA-*ζ*), KY425757 (*vosA* transcript variant 7; VosA-*η*), KY425758 (*vosA* transcript variant 8; VosA-*θ*), KY425759 (*velB* transcript variant 1; VelB), KY425760 (*velB* transcript variant 2; VelB), KY425761 (*velC* transcript variant 1; VelC) and KY425762 (*velC* transcript variant 2; VelC). The raw transcriptome sequence data and microarray gene expression raw data of *A. terreus* MUCL 38669 were submitted to the Sequence Read Archive (SRA) and Gene Expression Omnibus (GEO) databases of NCBI. The obtained accession numbers are PRJNA360953 (BioProject) and GSE93552 (GEO database).

## 3. Results

### 3.1. Strand-Specific Whole Transcriptome Sequencing and Refinement of Genome-Wide Gene Expression Analysis of A. terreus Strain MUCL 38669

The transcriptome RNA to be sequenced was obtained from the same samples used previously for whole genome gene expression study [19], from *Aspergillus* cultures that were exogenously fortified with butyrolactone I at 120 h post-inoculation. The extracted mRNA was pooled prior to the strand-specific sequencing performed in this study. This sequencing resulted in an approximate sequence coverage of 88 over the obtained transcriptome assembly of strain MUCL 38669, with good read sequence quality and indicates 89% of the annotated genes of strain NIH2624 to be expressed (the transcriptomic details are in Tables S1 and S2, Files S1 and S2 and Figure S1). The sequenced transcripts were also utilized to improve the reliability of the probes used previously for microarray gene expression study [19], where the probe design was based on the NIH2624 strain, being the only annotated *A. terreus* strain at that time. The strain MUCL 38669 was examined in the study due to its industrial importance and was observed to differ from strain NIH2624 at the nucleotide level, which led us to extract the more reliable probe signals through aligning the designed oligonucleotide probes with the obtained transcripts, as described in the Methods Section. This resulted in an approximate average of 65% of the transcripts to have a valid probe, which enabled us to perform more reliable differential gene expression analysis. The signal comparability amongst these extracted, valid probes is visualised in Figures S2 and S3.

### 3.2. The Members of the A. terreus Velvet Family

To our knowledge, *A. terreus* velvet family genes have not been previously studied. Only the *A. nidulans laeA* gene has been overexpressed in *A. terreus* strain ATCC 20542 leading to increased lovastatin production [39]. In this study, a BLASTP search at the protein level revealed only three genes with predicted velvet domains and the putative LaeA protein in *A. terreus* NIH2624 using *A. nidulans* velvet complex proteins VeA, VelB, VelC, VosA and LaeA as queries (AAD42946.1, ABQ17967.1, ABQ17968.1, ABI51618.1 and CBF88745.1, respectively). The obtained BLASTP results included the *A. terreus* orthologs for VeA (gene ATEG_00439), VelB (gene ATEG_04893), VosA (gene ATEG_03984) and LaeA (gene ATEG_00678) (Table 1).

#### 3.2.1. Revealing the *A. terreus velC* Gene

**Figure 2:**
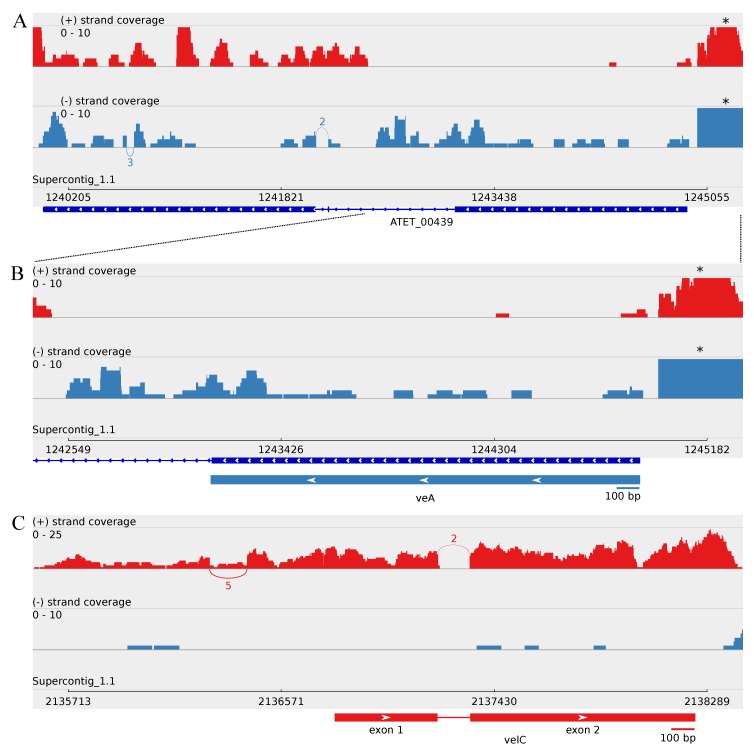
The transcript structures of *A. terreus* MUCL 38669 *veA* and *velC* orthologs. Sashimi plots of (**A**) the whole length of ATEG_00439 as annotated, (**B**) the predicted *veA* ortholog on 5${}^{\prime }$ of ATEG_00439 and (**C**) the revealed *velC* ortholog (between ATEG_00762 and ATEG_00763) describing the strand-specific alignment coverage of the read sequences of *A. terreus* MUCL 38669 over the corresponding genomic regions of supercontig_1.1 of NIH2624. The curved splice junctions represent the number of spliced reads indicating an intron at that specific location. The boxes represent the exons; the arrow heads indicate the genomic strand of the transcript; and the lines represent introns. The *veA* ORF prediction was obtained using the GENSCAN Web Server [71]. * These apparent coverage blocks consist of minor parts of the transcripts that align over the upstream region of ATEG_00446, indicating this genomic sequence region to be actually part of the upstream region of ATEG_00446 due to the similar transcription level and the corresponding profiles of blunt ends of the blocks (data not shown).

Since no ortholog for the VelC of *A. nidulans* was found amongst the predicted *A. terreus* NIH2624 proteins, a genomic BLASTN search using the *A. nidulans velC* gene sequence (EF540816.1) as a query was performed. The BLASTN search results revealed similarity to a genomic region between genes ATEG_00762 and ATEG_00763 of *A. terreus* NIH2624. A closer look at the obtained transcriptome sequence data of *A. terreus* strain MUCL 38669 revealed two transcripts of different lengths, 2545 bp and 2696 bp, on that specific location (Table 2 and Table S3). A further analysis of these transcripts revealed the occurrence of alternative splicing regarding the first intron, located upstream of the open reading frame (ORF), having no effect on the ORF start site (Figure 2, Table 2). Approximately 63% (five splice junctions) of the read coverage over this upstream intron region supports the presence of the intron (Table 2, Figure 2). At the protein level, the translated VelC is predicted to contain a velvet domain towards the C-terminus, and a BLASTP analysis revealed 53% identity with *A. nidulans* VelC protein (Table 1). Taken together, *A. terreus* MUCL 38669 contains a *velC* gene ortholog, as well.

#### 3.2.2. The Similarity amongst Velvet Complex Members of Some *Ascomycota* Fungi

**Figure 3:**
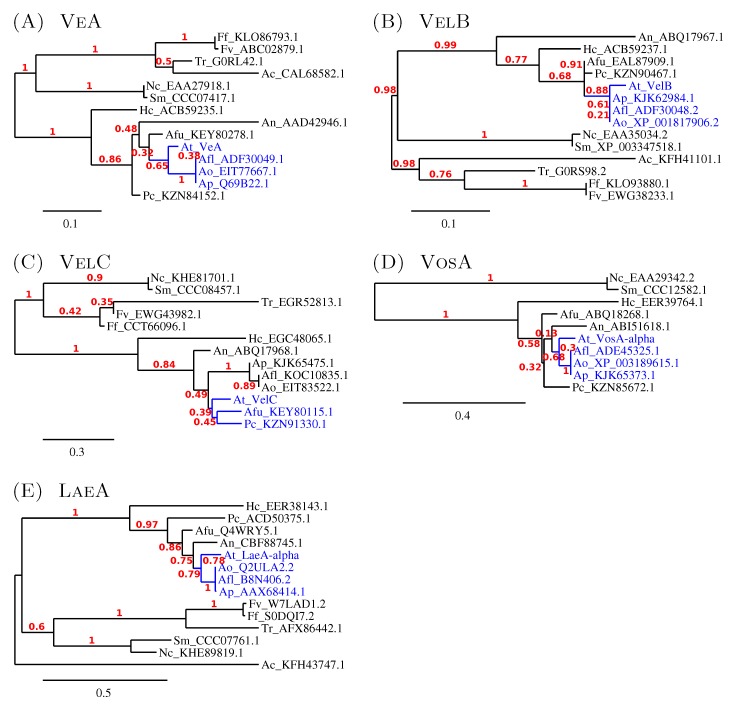
Phylogeny of velvet family members of several *Ascomycota* fungi. The separate phylogenetic trees consist of velvet proteins similar to (**A**) VeA, (**B**) VelB, (**C**) VelC, (**D**) VosA and (**E**) LaeA of *A. nidulans*. The protein sequences of the velvet members were obtained, aligned and phylogenetically grouped as described in the Methods. The scale bars represent the residue change per site, and the numbers indicate the probability of the suggested differentiation. No outgroup was used except for the LaeA phylogram. The organisms used in the phylograms: Ac, *Acremonium chrysogenum*; Afl, *Aspergillus flavus*; Afu, *Aspergillus fumigatus*; An, *Aspergillus nidulans*; Ao, *Aspergillus oryzae*; Ap, *Aspergillus parasiticus*; At, *Aspergillus terreus*; Ff; *Fusarium fujikuroi*; Fv, *Fusarium verticillioides*; Hc, *Histoplasma capsulatum*; Nc, *Neurospora crassa*; Pc, *Penicillium chrysogenum*; Sm, *Sordaria macrospora*; Tr, *Trichoderma reesei*. The protein sequences (except for At_VeA, At_VelB, At_VelC, At_VosA and At_LaeA) were obtained from the following databases: GenBank, EMBL, UniProtKB and NCBI RefSeq.

While searching for the putative velvet complex orthologs in *A. terreus*, intriguing similarity approximations between different *Aspergillus* species were revealed (Table 1). At the protein level, the VeA and VelC orthologs of *A. terreus* appear to be the most distinct velvet complex proteins in comparison to *A. nidulans* respective proteins, being 53% identical according to BLASTP search results, while VelB, VosA-*α* and LaeA-*α* have identities of 67%, 73% and 74%, respectively (Table 1). Regarding the *Aspergillus flavus* velvet family proteins, the *A. terreus* orthologs appear to be more similar, with 57%–87% identities. Especially the VeA and VelB orthologs are significantly closer to the respective orthologs of *A. flavus* being 70% and 85% identical at the protein level (Table 1). A phylogenetic analysis of the protein level similarity of the velvet family members amongst the *Ascomycete* species indicates the *A. nidulans* genes to be further diversified when compared with *A. terreus* orthologs, as well as the other included Aspergilli (*A. flavus*, *A. oryzae*, *A. fumigatus* and *A. parasiticus*). These Aspergilli gene orthologs, except for *A. fumigatus*, appear to form a more similar group diverging from the corresponding *A. nidulans* velvet family member (Figure 3). These phylogenetic trees are in good agreement with the obtained BLASTP alignment results.

### 3.3. Minor Updates of the A. terreus veA and velB Genes

The transcriptome sequencing results revealed different transcript structures than predicted for *A. terreus* NIH2624 regarding the velvet family genes *veA*, *velB*, *vosA* and *laeA*, as well. The transcript of *veA* ortholog appears to be significantly shorter than annotated for strain NIH2624, although being only partially covered in the transcriptome data. The first annotated intron on the 5${}^{\prime }$ end of *A. terreus* NIH2624 gene ATEG_00439 is however partially transcribed, revealing an earlier encoded stop codon. The 3${}^{\prime }$ end of the controversial ATEG_00439 gene is also transcribed, although being only partially covered and mainly over the antisense strand (Figure 2). The indicated genomic locations of these two genes were confirmed by GENSCAN analysis of the *A. terreus* NIH2624 sequence covering this genomic region. The resulting predicted ORF length for *A. terreus veA* gene ortholog is 1770 bp, having no introns in accordance with the transcriptome data (Figure 2, Table 2 and Table S3).

**Figure 4:**
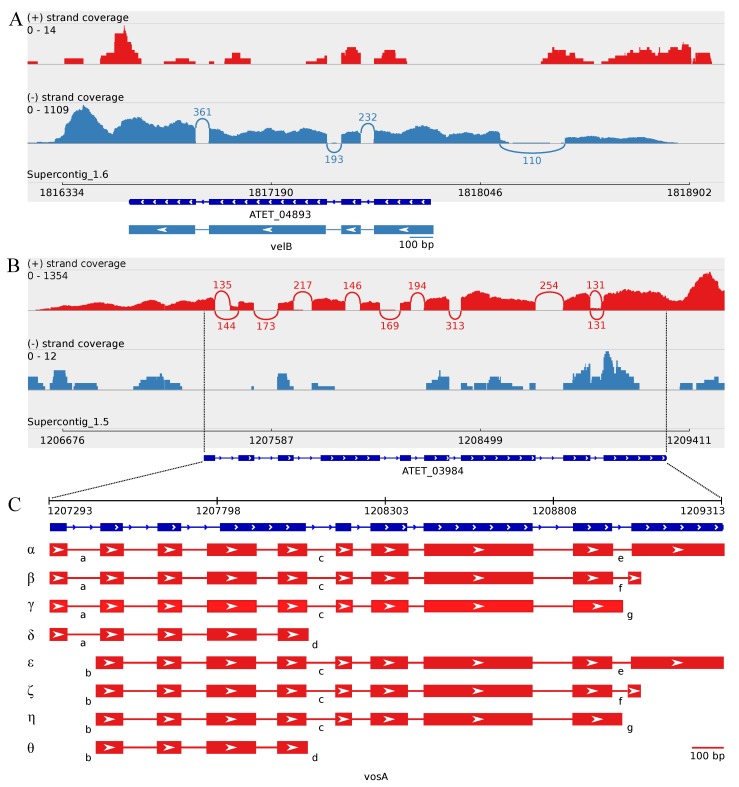
The transcript structure of *velB* ortholog and splice variants of *vosA* ortholog. Sashimi plots of (**A**) the *velB* ortholog and (**B**) the *vosA* ortholog describing the strand-specific alignment coverage of the read sequences of *A. terreus* MUCL 38669 over the corresponding genomic regions of NIH2624. The curved splice junctions represent the number of spliced reads, indicating an intron at that specific location. (**C**) An enlarged figure of the splice variants of the *vosA* ortholog as indicated by the Sashimi plot (**B**). The boxes represent the exons; the arrow heads indicate the genomic strand of the transcript; and the lines represent introns. The apparent alternative splicing of *vosA* ortholog occurs at three locations with the following approximate proportions of the number of indicative reads: a, 52%; b, 48%; c, 91%; d, 9%; e, 40%; f, 40%; and g, 20%; resulting in eight putative splice variants of *vosA*: alpha (*α*), beta (*β*), gamma (*γ*), delta (*δ*), epsilon (*ε*), zeta (*ζ*), eta (*η*) and theta (*θ*).

The transcript structure of the *velB* ortholog appears to indicate a slightly longer ORF than annotated for the NIH2624 strain, encoding an earlier start code leading to a 12 bp longer ORF (Table 2 and Table S3). Otherwise, the exon locations are the same as annotated for the strain NIH2624. The *velB* transcript has an upstream intron, which displays putative minor alternative splicing on the transcript level resulting in two transcripts with different lengths (Table 2), while the ORF length remains unchanged. Approximately 88% of the covering reads with 110 splice junctions indicate the presence of this upstream intron (Figure 4, Table 2).

### 3.4. The Transcriptome Sequencing Revealed Numerous Splice Variants for vosA and laeA Orthologs during Enhanced Butyrolactone I Biogenesis

#### 3.4.1. Splice Variants of the *A. terreus vosA* Ortholog

The *vosA* gene ortholog of *A. terreus* strain MUCL 38669 appears to have an extensive alternative splicing when butyrolactone I is exogenously added at 120 h post-inoculation. In total, there are eight possible splice variants (designated with alpha (*α*), beta (*β*), gamma (*γ*), delta (*δ*), epsilon (*ε*), zeta (*ζ*), eta (*η*) and theta (*θ*)) occurring under these growth conditions, as indicated by the transcript profile (Figure 4). The obtained transcripts of the MUCL 38669 strain revealed an additional intron inside the fourth exon of the predicted annotation of NIH2624 strain, leading to two separate exons (Table S3). Regarding the additional intron, there is a minor splice variant with an approximate 9% proportion encoding an early stop codon (Figure 4C(d)). The majority of the transcripts, with 169 splice junctions displaying a full-length intron, contains a significantly longer ORF (Figure 4 and Table 2).

Secondly, the more common splice variants are exclusively located at the 5${}^{\prime }$ and 3${}^{\prime }$ introns of the ORFs (Figure 4). The alternative splicing on the 5${}^{\prime }$ intron of the transcripts leads to a shift of the ORF Start site, resulting in the emergence of a putative upstream intron on the *vosA* variants *ε*, *ζ*, *η* and *θ* (Table S3). This upstream intron is slightly shorter than the corresponding intron inside the ORFs of the variants *α*, *β*, *γ* and *δ* and has also a minor occurrence of 135 splice junctions with an approximate proportion of 48% (Figure 4C(b)). As a result, the second exon in the *α*, *β*, *γ* and *δ* variants is 68 bp in length (Figure 4C(a)), while in the variants *ε*, *ζ*, *η* and *θ*, the first ORF exon is 82 bp in length (Figure 4C(b)). The approximate occurrence proportion of 52% with 144 splice junctions leads to an encoded first 5${}^{\prime }$ exon of 50 bp in length for the variants *α*, *β*, *γ* and *δ* (Figure 4C(a) and Table S3).

Regarding the 3${}^{\prime }$ ends of the full-length splice variants, *α*, *β*, *γ*, *ε*, *ζ* and *η*, there appears to be three different forms of splicing. The more common ones (*α* and *β*) have intron lengths of 59 bp and 48 bp, with indicative 131 splice junctions each (40% proportion), resulting in 3${}^{\prime }$ exons of 276 bp and 36 bp in length, respectively (Figure 4C(e,f) and Table S3). The third observed, but minor intron variant (with the approximate occurrence proportion of 20%) results in a shorter ORF with the last 3${}^{\prime }$ exon of length 147 bp (Figure 4C(g) and Table S3). Taken together, these depicted eight putative splicing combinations result in different ORF lengths, ranging from 387 bp to 1293 bp and the number of exons from 4–10, while the *vosA-α* and *vosA-β* appear to be the most common variants under these growth conditions (Table 2, Figure 4 and Table S3).

At the protein level, the putative VosA variants *α*, *β*, *γ*, *ε*, *ζ* and *η* contain the predicted velvet domain (pfam11754) of length 173 aa on their N-termini, whereas the variants *δ* and *θ* appear to contain only a partial velvet domain of 123 aa in length. The six longer variants are also predicted to contain an additional NADB_Rossmann superfamily domain (cl21454) on their C-termini, although being presumably partial at the C-termini (Figure S4).

#### 3.4.2. The Two Encoded Splice Variants of the *A. terreus laeA* Ortholog

**Figure 5:**
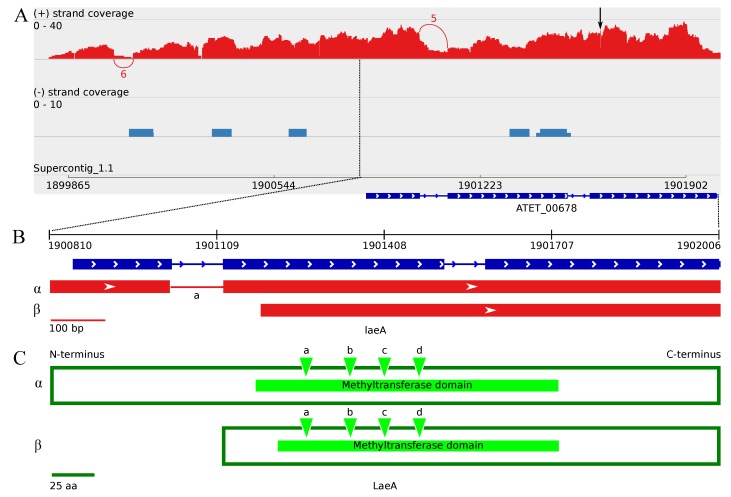
Splice variants and predicted protein domains of the regulative methyltransferase LaeA ortholog. Sashimi plot of (**A**) the *laeA* ortholog describing the strand-specific alignment coverage of the read sequences of *A. terreus* MUCL 38669 over the corresponding genomic region of supercontig _1.1 of NIH2624. The curved splice junctions represent the number of spliced reads indicating an intron at that specific location. The black arrow on top of the coverage profile indicates a 1-bp deletion at 1901621(+). (**B**) An enlarged figure of the ORF-level splice variants of the *laeA* ortholog as indicated by the Sashimi plot. The boxes represent the exons; the arrow heads indicate the strand of the transcript; and the lines represent introns. The apparent alternative splicing that leads to two different ORFs occurs at the second transcriptional intron (a). Approximately 36% of the covering reads support the occurrence of the intron, resulting in a *laeA-α* splice variant. Approximately 64% of the covering reads that support the absence of the intron result in a *laeA-β* variant. (**C**) A schematic description of the encoded protein variants (*α* and *β*) of the LaeA ortholog with the predicted methyltransferase domain (pfam13489). The triangles indicate the predicted SAM-binding sites (a: seven amino acid residues; b: 2 aa; c: 3 aa; d: 1 aa).

The transcriptome data revealed four transcriptomic splice variants for the known global regulator *laeA* ortholog, as well, resulting to two ORFs of different lengths, 1104 bp (*laeA-α*) and 822 bp (*laeA-β*). Both of the splice variants contain a deletion (1 bp) when compared to the annotated ORF sequence of the NIH2624 strain (located at supercontig_1.1: 1901621(+)). The subsequent frameshift suppresses the upstream frameshift, which is caused by the absence of the upstream 3${}^{\prime }$ intron, the presence of which has been annotated for the strain NIH2624 (Figure 5). The shorter variant, *laeA-β*, appears to be slightly more prevalent under these growth conditions with an approximate 64% proportion, supporting the absence of the remaining intron, located at the 5${}^{\prime }$ region of the *laeA-α* variant (Table S3). Both of the splice variants appear to have an intron on the upstream region, which reveals putative alternative splicing, as well, without any effect on the ORF length. The presence of this upstream intron is supported by an approximate 67% proportion of reads displaying splice junctions (Figure 5 and Table S3). In conclusion, the most common splice variant appears to be *laeA-β* with an upstream intron. On protein level, both of the two splice variants (LaeA-*α* and LaeA-*β*) contain the putative methyltransferase domain (pfam13489) with all 13 predicted S-adenosylmethionine (SAM)-binding sites present (Figure 5C).

### 3.5. Gene Expression Profiles and Pooled Transcript Accumulation of the Velvet Family, laeA and Conidiation Core Regulators under Butyrolactone I Influence

**Figure 6:**
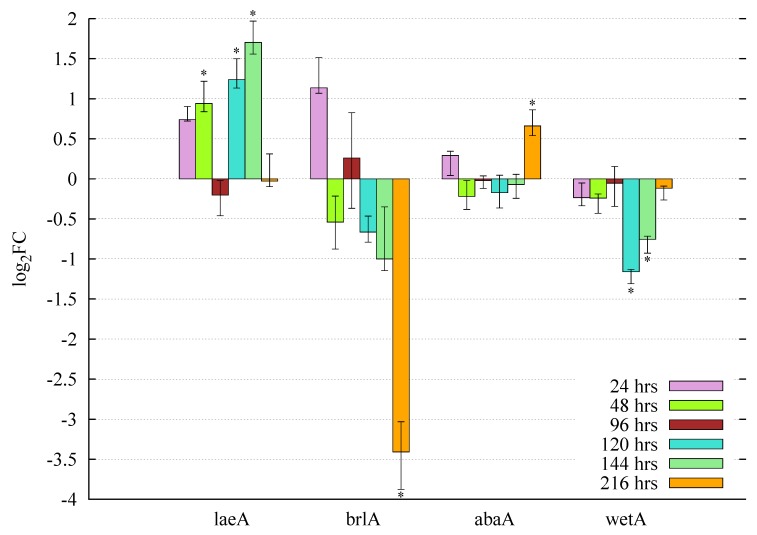
Butyrolactone I upregulates the *A. terreus laeA* ortholog and appears to play a role in the expression of the core conidiation regulator orthologs *brlA*, *abaA* and *wetA*. Exogenous butyrolactone I was added to the submerged culture of *A. terreus* MUCL 38669 at 24 h p.i. to the final concentration of 100 nM. Samples were analysed for gene expression using custom-designed microarrays as described in the Methods. The bars represent the median values of the log${}_{2}$FC of treated samples (butyrolactone I was added) vs. control samples (no butyrolactone I added), and the error bars represent the maxima and minima of these log${}_{2}$FC values. * At least one of the four technical replicates indicates statistically-significant differential expression in the three biological replicates (adjusted *p*-value ≤0.05 and |log${}_{2}$FC| ≥0.5).

In order to investigate whether the exogenously-added butyrolactone I affects the gene expression of the velvet family members and the related core regulators of conidiation, the obtained filtered microarray gene expression results were examined. The amount of valid gene expression results is limited due to the reliability improving probe validity assessment conducted in this study. However, the reliable microarray gene expression results display statistically-significant gene expression profiles for the global regulator ortholog *laeA* and the core conidiation regulator orthologs *brlA* (ATEG_05140), *abaA* (ATEG_02625) and *wetA* (ATEG_03205) (Figure 6).

#### 3.5.1. Butyrolactone I Upregulates the Global Regulator *laeA* Ortholog

The exogenous butyrolactone I addition reveals two intriguing and separately occurring increased periods of *laeA* expression when added at the early growth phase, 24 h post-inoculation (p.i.). Specifically, the upregulation occurs at least during the early growth phase (48 h p.i.) and during the late growth phase (120 h and 144 h p.i.) with statistically-significant fold change values (0.5< log${}_{2}$FC <2 with *p*-value <0.05) (Figure 6). The upregulation of *laeA* is also statistically significant and presumably direct, as well, when butyrolactone I is added during the middle growth phase, i.e., at 96 h post-inoculation, while the addition during the beginning of the late growth phase (at 120 h p.i.) appears not to have any significant effect (Figure S5).

#### 3.5.2. Butyrolactone I Plays a Role in the Control of the Core Regulators of Conidiation

The examined microarray results reveal also interesting gene expression profiles for the orthologs of *A. nidulans* core regulators of conidiation, *brlA*, *abaA* and *wetA* with residue identities 75%, 62% and 61%, respectively (Figure 6, Table S4). The exogenous addition of butyrolactone I at 24 h post-inoculation displays significant transcriptional repression of the transcription factor *brlA*, especially at the end of the late growth phase, 216 h post-inoculation (−4< log${}_{2}$FC <−3). In contrast, the regulator *abaA* shows stable non-differential gene expression in comparison to the untreated culture until the end of late growth phase when it is statistically-significantly upregulated (at 216 h p.i., 0.5< log${}_{2}$FC <1). The third transcriptional regulator *wetA* is significantly repressed during the late growth phase (at 120 and 144 h p.i., −1.5< log${}_{2}$FC <−0.5), while at the end of this phase (216 h p.i.), the gene expression is no longer downregulated (Figure 6). When exogenous butyrolactone I is added during the middle (96 h p.i.) or at the beginning of the late growth phase (120 h p.i.) (Figure S5), the gene expression profiles are basically very similar, but less significant in comparison with the profiles obtained with the earlier addition time point (24 h p.i.) (Figure 6).

#### 3.5.3. Accumulation Levels of the Pooled, Sequenced Transcripts

The pooled transcriptome RNA to be sequenced in this study consisted of six RNA samples (taken at 24, 48, 96, 120, 144 and 216 h p.i.) where butyrolactone I had been added at 120 h p.i., restricting the extent and usability of the calculated and normalised amounts of the obtained transcripts, represented as fragments per kilobase of exon per million reads mapped (FPKM). However, the pooled accumulation levels of the velvet family orthologs can be divided into three approximate accumulation groups, high level of *vosA* and *velB* (110 and 120 of pooled FPKM, respectively), active accumulation of *laeA* (6.8 of pooled FPKM) and low level of *veA* (0.83 of pooled FPKM; partial coverage). The obtained transcript read coverage medians of the pooled RNA (represented as pooled counts) within a window size of 25 bp and applied over the genome show also similar division into accumulation groups as the pooled FPKM values for the *vosA*, *velB*, *laeA* and *veA* orthologs, including also the *velC* ortholog. The maximum values of these pooled counts values on the sense strand are 73 for *vosA*, 62 for *velB*, 2.1 for *laeA*, 1.3 for *velC* and 0.45 for *veA*, indicating the *velC* ortholog to be actively expressed similarly to the *laeA* ortholog although on a slightly lower level (Table 3). The pooled counts’ maximum values regarding the antisense strand for the whole velvet family genes are very low, indicating no antisense repression at the converged sample time points. Taken together, the velvet family gene orthologs *velB*, *velC*, *vosA* and *laeA* appear to be actively expressed during or at some of the six sampling time points of the nine day-long submerged culturing, while the *veA* ortholog is presumably inactive in these growth conditions.

The core regulators of conidiation, the *brlA*, *abaA* and *wetA* orthologs, show increasing amounts of accumulated transcripts of pooled RNA samples (1.5, 7.0 and 13 of pooled FPKM, respectively). The transcripts of α,α-trehalose-phosphate synthase 1, *tpsA* (ATEG_03327) display a high transcript accumulation of pooled RNA samples with the pooled FPKM value of 270 (Table 3). The sequenced transcripts of *abaA*, *wetA* and *tpsA* revealed no structural differences and, thus, display the same gene and ORF lengths in comparison to the corresponding gene annotations of the strain NIH2624 (Figure S6 and Table S4).

## 4. Discussion

In *A. terreus*, butyrolactone I has been demonstrated to induce features that suggest a regulative role of the quorum-sensing phenomenon. These include autoinduction, an increase in secondary metabolite biosynthesis (lovastatin and sulochrin) and sporulation [18,19,20]. However, the regulatory role of butyrolactone I on the velvet family genes has not been previously reported. Amongst *Aspergillus* species, especially *A. nidulans*, the velvet family members have been connected to the regulation of secondary metabolism and cellular differentiation, including sporulation (Figure 1) [31,32,33,37,38].

### 4.1. Involvement of Butyrolactone I in the Control of Asexual Sporulation through LaeA

The key regulatory genes of conidiation, *brlA*, *abaA* and *wetA*, have been mainly studied in *A. nidulans* on static, aerial conditions. The first conidiation-specific fungal cell differentiation phase, vesicle and metulae formation is positively regulated by BrlA. The subsequent phialide emergence is induced by AbaA, followed by conidiophore maturation and increase in spore viability, which is controlled by WetA in a positive manner [24,25,26,27,28,29,34,84]. In *A. terreus*, exogenous addition of butyrolactone I has been observed to increase sporulation and hyphal branching [18]. In our study, the transcription factor *brlA* is shown to be downregulated especially at the late growth phase (216 h p.i.), while *abaA* revealed statistically-significant upregulation at the same time point as a result of exogenous butyrolactone I addition at 24 h post-inoculation in submerged culture. *wetA* is shown to be significantly downregulated starting from the middle growth phase proceeding towards the end of late growth phase, i.e., at two time points between 96 h and 216 h post-inoculation, presumably enabling the hyphal branching to continue, followed by hyphae dying and lysis. Moreover, the cease of downregulation when the end of late growth phase is reached (at 216 h p.i.) indicates that the conidial maturation has begun, in agreement with the increase in phialide emergence as suggested by the upregulation of *abaA* at this time point. The significant downregulation and low, pooled transcript accumulation level of *brlA* may indicate the initiative phase of sporulation, i.e., vesicle and metulae budding, to occur during the interval of 72 h between the sampling time points of late growth phase, prior to 216 h post inoculation. Alternatively, the vesicle and metulae development might be bypassed as has been observed for *A. nidulans* under specific submerged conditions [85]. The expression of *A. nidulans* trehalose biogenesis transcripts (*tpsA*) has been reported to increase during the conidiation phase, in accordance with the amount present in the conidia, and to be involved in the improvement of the spore viability [30,31,32,33]. The observed high, pooled accumulation level of *tpsA* transcripts in this study (Table 3 and Figure S6) supports the indications of the conidia development under these growth conditions. In addition, *laeA* displayed upregulation during the early growth phase (48 h p.i.), as well as during the late growth phase (120 and 144 h p.i.) when butyrolactone I was added in the onset of the early growth phase (24 h p.i.) (Figure 6). All of this indicates *laeA* to be positively regulated prior to the conidiation stage when butyrolactone I was added at the early phase. In *A. nidulans*, LaeA has been observed to control *abaA* expression on transcriptional level in a positive manner, while *brlA* was less affected [32]. Taken together, this supports the reported sporulation-inducive role for butyrolactone I [18] and suggests the regulation to occur through LaeA.

### 4.2. The Divergent Variants of the Global Regulator laeA under Submerged Culture

To our knowledge, no velvet family genes have been studied in *A. terreus*, except for *laeA*. The *A. nidulans laeA* gene was overexpressed in *A. terreus* and was observed to increase lovastatin production, indicating LaeA to control secondary metabolism in *A. terreus*, as well [39]. Exogenous addition of butyrolactone I has also been reported to increase secondary metabolite production (lovastatin, sulochrin and butyrolactone I itself) in *A. terreus* [18,19,20]. In this study, *laeA* is observed to be upregulated by exogenous addition of butyrolactone I (Figure 6), indicating the previously observed positive control of secondary metabolism to occur through LaeA.

Intriguingly, *laeA* also displays alternative splicing leading to two variants, *α* and *β*, with different ORF lengths. On the encoded protein level, these variants differ in their N-terminus length, while the predicted length of the methyltransferase domain and the SAM-binding sites are principally preserved (Figure 5). Since no actual protein level information regarding the *A. terreus* velvet complex is available, one can only speculate whether these observed splice variants have any functional differences. In *A. nidulans*, LaeA has been reported to control the secondary metabolite production and sporulation when forming a heterotrimeric complex with VeA and VelB in dark growth conditions. Specifically, the N-terminus of LaeA was suggested to interact with the C-terminus of VeA, while the N-termini of VeA and VelB were indicated to interact with each other in order to form a functional trimeric heterocomplex [37]. In this study, both *laeA* and *velB* pooled transcripts appeared to be numerous, whereas the *veA* transcript was only partially covered (Table 3, Figure 2, Figure 4 and Figure 5). The supposed interaction between LaeA and VeA may thus be quite low, which would indicate the secondary metabolism and sexual sporulation to be absent, as well. However, one can speculate about the occurrence of at least two additional options regarding the protein interaction between LaeA and VeA : (i) the *veA* transcript may have been temporarily expressed during the late growth phase, similarly to the hypothesised transcript accumulation pattern of *brlA* (between 144 h and 216 h p.i.), leading to the inducement of secondary metabolism and sporulation, or (ii) the secondary metabolism and sporulation was induced by LaeA in an alternative manner related to the observed alternative splicing of *laeA*. The *laeA-β* splice variant appears to be more frequent (approximately 64% of *laeA* transcripts) and encodes the predicted methyltransferase-related domain, but may be disabled to interact with VeA due to the lack of the encoded full-length N-terminus. This would also explain the observed contradictory effects of butyrolactone I addition to increase both asexual sporulation, as well as secondary metabolism of *A. terreus* under submerged culture [18,19]. Taking the obtained gene expression profile and the pooled accumulated splice variants of *laeA* into consideration, the distribution of upregulated splice variants in the sampling time points remains unspecific. Deeper and more comprehensive protein level studies would be required to further enlighten and confirm the role of the observed alternative splicing of *laeA*.

### 4.3. Accumulation of vosA, velB and velC Pooled Transcripts under Influence of Enhanced Butyrolactone I Biogenesis

In *A. nidulans*, one of the regulators of conidiation, AbaA, has been suggested to activate both *velB* and *vosA* in phialides through binding to the promoter region [31,32,33]. In this study, *vosA* and *velB* transcripts appear to be accumulated on a higher level in the pooled samples than *laeA*, *velC* and *veA* throughout the submerged culture when butyrolactone I was added during the late growth phase (Table 3). Accordingly, *abaA* was observed to be upregulated when butyrolactone I was also added during the late growth phase (Figure S5). In *A. nidulans*, VelB and VosA have been observed to play various roles regarding the fungal differentiation phases, including vegetative growth, phialide emergence and conidia maturation (Figure 1) [31,32,33]. In hyphae, the VelB homodimeric complex is suggested to activate conidiation, while the VosA homodimeric complex is suggested to repress conidiation. In addition, a heterodimeric VelB-VosA complex has been reported to repress *brlA* expression in phialides, thus performing negative feedback regulation of conidiation, while in conidia, the complex is suggested to activate *wetA* and ensure spore viability along with TpsA activity [31,32,33,37]. In the *A. terreus* submerged culture of this study, *brlA* was observed to be negatively regulated at the same time point of late growth phase, as *abaA* was positively regulated (Figure 6 and Figure S5), supporting the hypothesis of *brlA* being active between the transcriptional snapshots of 144 h and 216 h post-inoculation. The VelB-VosA complex has also been reported to be under post-translational regulation by LaeA in a light-dependent manner. The complex was observed to be repressed by LaeA in light, enabling the asexual differentiation in *A. nidulans* [32]. In accordance, *laeA* showed no upregulation at 216 h post-inoculation, i.e., the last sampling time point of late growth phase (Figure 6 and Figure S5), indicating the VelB-VosA complex to be released, enabling the spore maturation and viability to proceed as indicated by the observed upregulation of *abaA* and the release of *wetA* repression. Altogether, the abundant diversity of the proposed functions for both VelB and VosA is in good accordance with the high pooled transcript accumulation observed in this study and is also in accordance with the presented suggestions of the cellular development of *A. terreus* as a result of induced butyrolactone I biogenesis. To confirm these results, further protein level and time point-specific studies would be required.

To our knowledge, the functional role of the VelC of the velvet complex members has been very scarcely studied. Recently, VelC has been suggested to be involved in the control of both sexual and asexual sporulation processes by activating the sexual development while indirectly repressing the asexual development in *A. nidulans* [38]. VelC has also been observed to bind to VosA and be expressed during vegetative growth, as well as in the early phase of sexual sporulation [38]. The low pooled transcript accumulation level of *velC*, while being higher than *veA*, may be due to the presumably long vegetative growth phase during submerged culture as indicated by the gene expression profiles of *brlA*, *abaA* and *wetA* (Table 3, Figure 6 and Figure S5).

### 4.4. Upstream Splice Variants of vosA, velC and laeA under the Influence of Butyrolactone I

The accumulation of VelB and VosA at the translational level is suggested to be uncorrelated with transcriptional expression level during both asexual and sexual development phases in *A. nidulans*, while at the protein level, LaeA was reported to release the repressional function of the complex in light, in order to enable asexual sporulation [32]. This divergence between transcriptional and translational accumulation indicates regulation at the translational level. In this study, the obtained transcript sequences of *laeA*, *velB* and *velC* revealed an upstream intron that revealed no effect on the predicted ORF length of each of the genes. Of these, introns upstream of the *velC* and *laeA* ORFs show also distinctive alternative splicing (Figure 2C and Figure 5), suggesting a role for translational modification. Regarding *vosA* and *laeA* transcripts, there is also alternative splicing affecting the encoded ORF length (Figure 4 and Figure 5). In *A. nidulans*, *vosA* has been reported to express two separate variants with different transcript and protein lengths in the early vegetative growth phase [31]. However, no further studies to verify these variants have been reported to our knowledge. These transcriptional variants may correspond to the splice variants of *vosA-α* versus *vosA-δ* or *vosA-ϵ* versus *vosA-θ* of *A. terreus*, having notable length differences between the ORFs observed in this study. The splice variants might be involved in the formation of diverse velvet protein dimer and trimer complexes in a post-transcriptionally regulative manner. In order to verify the presence of these alternative upstream splice junctions and their potential translation regulative role and implicated functions, further studies at the protein level will be essential.

## 5. Conclusions

In this study, we revealed several transcriptional updates of the velvet family genes and the global regulator *laeA* in *A. terreus* strain MUCL 38669 and discovered the genomic locus of the velvet family member *velC*. The observed splice variants of *vosA* and *laeA* that revealed different ORF lengths invoke intriguing hypotheses for further studies, whether the differences are present at protein level and have any functional influence. Butyrolactone I was also observed to rather directly upregulate *laeA*, the regulator involved in the secondary metabolism and sporulation control, in accordance with the suggested role for butyrolactone I as a quorum-sensing molecule in *A. terreus*, and thus, emphasises the necessity for further studies to specify the role of butyrolactone I in the development of this fungus.

## Figures and Tables

**Table 1:** Residue identity ${}^{a}$ of *A. terreus* velvet family members to *A. nidulans*, *A. flavus* and *A. fumigatus* orthologs ${}^{b}$.

*A. terreus*		*A. nidulans*		*A. flavus*		*A. fumigatus*	
Gene ${}^{c}$	Length (aa)	ID%	Accession	ID%	Accession	ID%	Accession
VeA ${}^{d}$	589	53	AAD42946.1	70	ADF30049.1	67	KEY80278.1
VelB	360	67	ABQ17967.1	85	ADF30048.2	82	EAL87909.1
VelC	441	53	ABQ17968.1	57	KOC10835.1	53	KEY80115.1
VosA-*α*	430	73	ABI51618.1	81	KOC10835.1	78	ABQ18268.1
VosA-*β*	350	75	ABI51618.1	82	KOC10835.1	80	ABQ18268.1
VosA-*γ*	348	75	ABI51618.1	83	KOC10835.1	80	ABQ18268.1
VosA-*δ*	140	79	ABI51618.1	87	KOC10835.1	88	ABQ18268.1
VosA-*ϵ*	418	74	ABI51618.1	82	KOC10835.1	78	ABQ18268.1
VosA-*ζ*	338	76	ABI51618.1	84	KOC10835.1	80	ABQ18268.1
VosA-*η*	336	76	ABI51618.1	84	KOC10835.1	80	ABQ18268.1
VosA-*θ*	128	83	ABI51618.1	91	KOC10835.1	88	ABQ18268.1
LaeA-*α*	367	74	CBF88745.1	80	B8N406 *^e^*	75	Q4WRY5
		NA	NA	87	EED56057.1 ${}^{f}$	NA	NA
LaeA-*β*	273	85	CBF88745.1	87	B8N406 *^e^*	84	Q4WRY5
		NA	NA	87	EED56057.1 ${}^{f}$	NA	NA

${}^{a}$ The residue identity percentage values were obtained using BLASTP [81]; ${}^{b}$ orthologous protein sequences were obtained from GenBank or UniProtKB; ${}^{c}$ translated on the basis of sequenced transcripts’ ORFs using the ExPASy Translate tool [74]; ${}^{d}$ translated on the basis of predicted ORF by the GENSCAN Web Server [71]; *^e^* 369 aa in length; ${}^{f}$ 282 aa in length; NA: not available.

**Table 2:** Sequenced transcript details of the *A. terreus* MUCL 38669 velvet family with splice variants.

	ORF	Gene	Transcript	Number of	Upstream	Approximate Occurrence
Gene	Length (bp)	Length (bp) ${}^{a}$	Length (bp) ${}^{b}$	Exons (Introns)	Intron	of the Upstream Intron (%)
*veA*	1770 ${}^{c}$	1770 ${}^{c}$	NA	1 (0) ${}^{c}$	NA	NA
*velB*	1083	1250	3432 ${}^{d}$	4 (4)	Yes	88 *^e^*
*velB*	1083	1250	3220 ${}^{d}$	4 (3)	No	-
*velC*	1326	1453	2545	2 (2)	Yes	63 *^e^*
*velC*	1326	1453	2696	2 (1)	No	-
*vosA-α*	1293	2021	2621	10 (9)	No	-
*vosA-β*	1053	1770	2632	10 (9)	No	-
*vosA-γ*	1047	1716	2680	9 (8)	No	-
*vosA-δ*	423	772	1429	5 (4)	No	-
*vosA-ϵ*	1257	1884	2658	9 (9)	Yes	48 ${}^{f}$
*vosA-ζ*	1017	1633	2669	9 (9)	Yes	48 ${}^{f}$
*vosA-η*	1011	1579	2717	8 (8)	Yes	48 ${}^{f}$
*vosA-θ*	387	635	1466	4 (4)	Yes	48 ${}^{f}$
*laeA-α*	1104	1196	2745	2 (2)	Yes	67 *^e^*
*laeA-α*	1104	1196	2808	2 (1)	No	-
*laeA-β*	822	822	2837	1 (1)	Yes	67 *^e^*
*laeA-β*	822	822	2900	1 (0)	No	-

${}^{a}$ Based on the genomic region of strain NIH2624 between 5${}^{\prime }$ and 3${}^{\prime }$ of the corresponding gene; ${}^{b}$ derived from the corresponding sequenced transcript of strain MUCL 38669, including sequenced up- and down-stream regions with upstream introns if present; ${}^{c}$ predicted with GENSCAN Web Server [71]; in accordance with the obtained partial transcript; ${}^{d}$ the sequenced transcript covers also gene ATEG_04892; *^e^* number of splice junctions/sum of read coverage median over the intron region and splice junctions; ${}^{f}$ number of the alternative splice junctions (135)/sum of both splice junctions; NA: not available.

**Table 3:** Pooled transcript accumulation of the velvet family members and core regulators of conidiation under enhanced butyrolactone I biogenesis ${}^{a}$.

Gene	Pooled ${}^{a}$ FPKM	Pooled Counts Max ${}^{b}$		Pooled Coverage Max ${}^{c}$	
		Sense	Antisense	Sense	Antisense
*veA*	0.83	0.45	0.057	8	1
*velB*	120	62	0.64	1109 ${}^{d}$	14
*velC*	NA	1.3	0.062	25 ${}^{d}$	1
*vosA-α*	110	73	0.66	1317 ${}^{d}$	12
*laeA-α*	6.8	2.1	0.12	40 ${}^{d}$	2
*brlA*	1.5	0.78	0.00	14	0
*abaA*	7.0	2.9	0.11	50 ${}^{d}$	2
*wetA*	13	3.8	0.25	70 ${}^{d}$	4
*tpsA*	270	77	0.98	1461 ${}^{d}$	16

${}^{a}$ Exogenous butyrolactone I was added at 120 h p.i. and the extracted RNA of samples taken at 24, 48, 96, 120, 144, 216 h p.i. was pooled prior to sequencing; ${}^{b}$ represents coverage medians of pooled RNA samples with a 25-bp window over the NIH2624 genome; ${}^{c}$ represents the number of overlapping nucleotides at one nucleotide site of the transcript obtained from the pooled RNA samples; ${}^{d}$ complete coverage; NA: not available.

## Supplementary Materials

The following are available online at www.mdpi.com/2076-2607/5/1/12/s1: Table S1: Information of the sequence quality. Table S2: Facts of the assembled transcriptome. Table S3: The ORF and intron locations of velvet family members and *laeA* transcripts. Table S4: Transcript details of *brlA, abaA, wetA* and *tpsA*. Figure S1: Distribution of the transcript accumulation (FPKM) of pooled RNA samples over the quantiles of the number of sequenced gene transcripts. Figure S2: Gene expression data in quartiles after normalisation. Figure S3: Gene expression data in quartiles after normalisation and filtering. Figure S4: Alignment of translated VosA splice variants with predicted domain regions. Figure S5: Gene expression profiles when butyrolactone I was added at (A) 96 h or (B) 120 h p.i. Figure S6: Sashimi plots of *brlA, abaA, wetA* and *tpsA*. File S1: Supplementary Methods, transcriptome and microarray raw data quality evaluation. File S2: A list of the normalised, mapped transcript quantities of the pooled RNA samples, as FPKM values, calculated over the annotated genes of strain NIH2624.

## Abbreviations

The following abbreviations are used in this manuscript:

FPKM	Fragments per kilobase of exon per million reads mapped
ORF	open reading frame
p.i.	post inoculation
FC	fold change
