# Supplementary material for: Transcriptomic Complexity of Aspergillus terreus Velvet Gene Family under the Influence of Butyrolactone I
Source: Microorganisms. 2017 Mar 14;5(1):12. doi: 10.3390/microorganisms5010012 (PMC5374389; doi:10.3390/microorganisms5010012)
Supplement: Supplementary file 1 [file microorganisms-05-00012-s001.zip › supplementary/Figure_S1.pdf]

Figure S1. Distribution of the transcript accumulation (FPKM) of pooled RNA samples over the quantiles of the number of sequenced gene transcripts. [a, b]

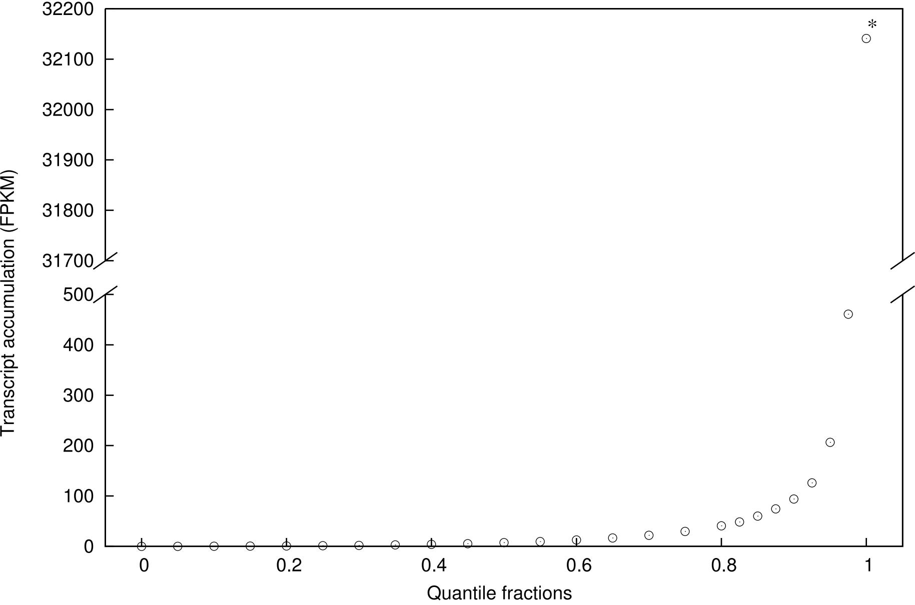

[a] *R software, function "quantile" was used*

[b] *Total amount of sequenced transcripts was 633388 FPKM*

** 5 % (32141 FPKM) of total amount of transcripts*
