# Supplementary material for: Transcriptomic Complexity of Aspergillus terreus Velvet Gene Family under the Influence of Butyrolactone I
Source: Microorganisms. 2017 Mar 14;5(1):12. doi: 10.3390/microorganisms5010012 (PMC5374389; doi:10.3390/microorganisms5010012)

Figure S3. Gene expression data in quartiles after normalisation and filtering

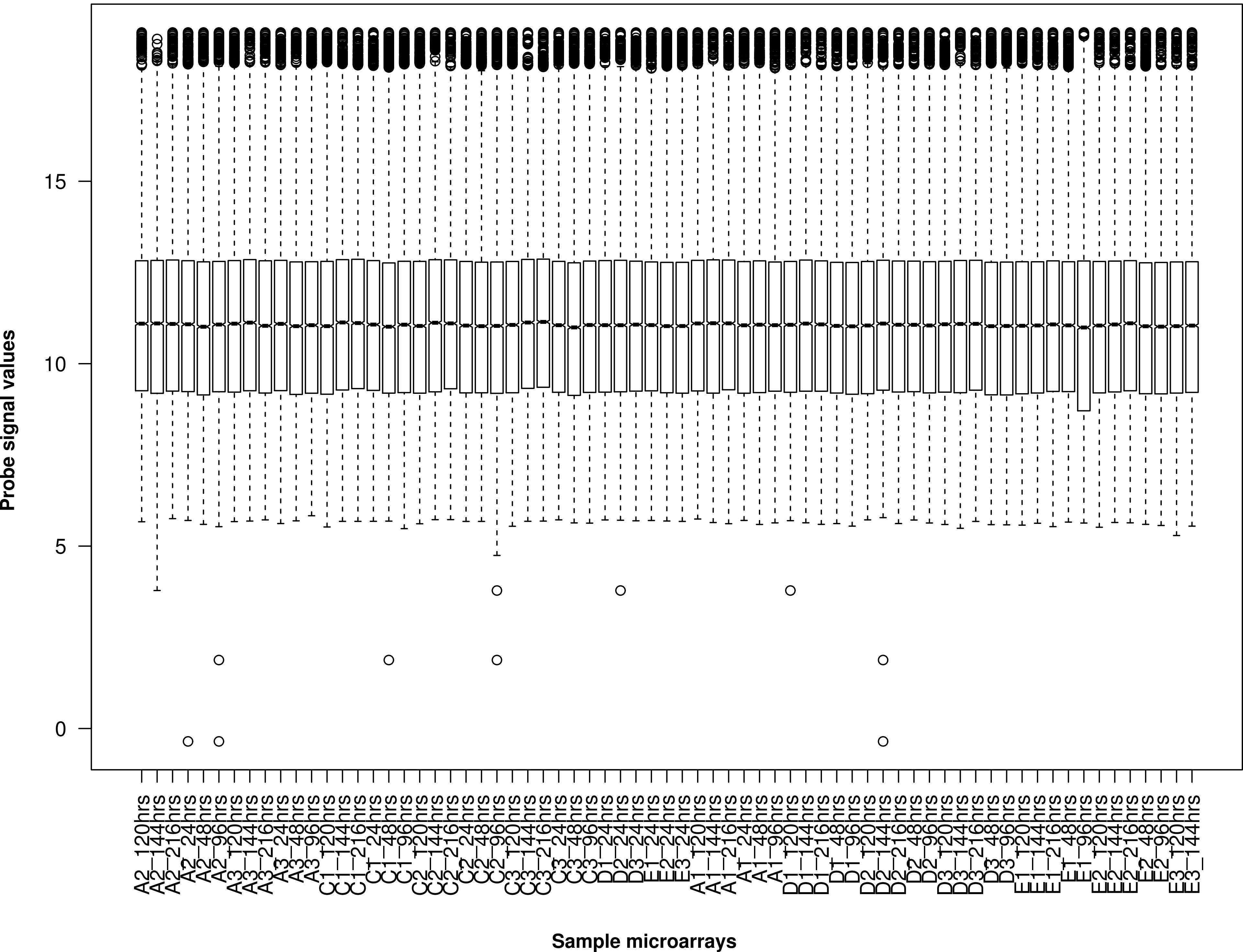

Supplement: Supplementary file 1 [file microorganisms-05-00012-s001.zip › supplementary/Figure_S3.pdf]
