# Supplementary material for: Transcriptomic Complexity of Aspergillus terreus Velvet Gene Family under the Influence of Butyrolactone I
Source: Microorganisms. 2017 Mar 14;5(1):12. doi: 10.3390/microorganisms5010012 (PMC5374389; doi:10.3390/microorganisms5010012)
Supplement: Supplementary file 1 [file microorganisms-05-00012-s001.zip › supplementary/Figure_S4.pdf]

Figure S4. Multiple sequence alignment (CLUSTAL 0(1.2.2)) of *A. terreus* MUCL38669 VosA splice variants with predicted protein domains as indicated.

```
                                         |            pfam11754 Velvet
                                         |___________________________________________

VosA-alpha|430aa        MSASTFSDASLARDPQSSEFDLIIRQQPTRARVAGGKEKERKPVDPPPIVQIRVKEDGTY    60
VosA-beta|350aa         MSASTFSDASLARDPQSSEFDLIIRQQPTRARVAGGKEKERKPVDPPPIVQIRVKEDGTY    60
VosA-gamma|348aa        MSASTFSDASLARDPQSSEFDLIIRQQPTRARVAGGKEKERKPVDPPPIVQIRVKEDGTY    60
VosA-delta|140aa        MSASTFSDASLARDPQSSEFDLIIRQQPTRARVAGGKEKERKPVDPPPIVQIRVKEDGTY    60
VosA-epsilon|418aa      ------------MLSPSSEFDLIIRQQPTRARVAGGKEKERKPVDPPPIVQIRVKEDGTY    48
VosA-zeta|338aa         ------------MLSPSSEFDLIIRQQPTRARVAGGKEKERKPVDPPPIVQIRVKEDGTY    48
VosA-eta|336aa          ------------MLSPSSEFDLIIRQQPTRARVAGGKEKERKPVDPPPIVQIRVKEDGTY    48
VosA-theta|128aa        ------------MLSPSSEFDLIIRQQPTRARVAGGKEKERKPVDPPPIVQIRVKEDGTY    48
                                        ********************************************

                                          pfam11754 Velvet
                        ____________________________________________________________

VosA-alpha|430aa        LASHYLQSPYYFMCCSLYDATEDTPVPVPPSTALTGTLVSSLHRLKDVDNTDGGFFVFGD    120
VosA-beta|350aa         LASHYLQSPYYFMCCSLYDATEDTPVPVPPSTALTGTLVSSLHRLKDVDNTDGGFFVFGD    120
VosA-gamma|348aa        LASHYLQSPYYFMCCSLYDATEDTPVPVPPSTALTGTLVSSLHRLKDVDNTDGGFFVFGD    120
VosA-delta|140aa        LASHYLQSPYYFMCCSLYDATEDTPVPVPPSTALTGTLVSSLHRLKDVDNTDGGFFVFGD    120
VosA-epsilon|418aa      LASHYLQSPYYFMCCSLYDATEDTPVPVPPSTALTGTLVSSLHRLKDVDNTDGGFFVFGD    108
VosA-zeta|338aa         LASHYLQSPYYFMCCSLYDATEDTPVPVPPSTALTGTLVSSLHRLKDVDNTDGGFFVFGD    108
VosA-eta|336aa          LASHYLQSPYYFMCCSLYDATEDTPVPVPPSTALTGTLVSSLHRLKDVDNTDGGFFVFGD    108
VosA-theta|128aa        LASHYLQSPYYFMCCSLYDATEDTPVPVPPSTALTGTLVSSLHRLKDVDNTDGGFFVFGD    108
                        ************************************************************

                                          pfam11754 Velvet
                        ____________________________________________________________

VosA-alpha|430aa        LSVKIEGDFRLKFTLFEMRKEMVYYLKSIISERFTVSPPKSFPGMAESTFLSRSFADQGV    180
VosA-beta|350aa         LSVKIEGDFRLKFTLFEMRKEMVYYLKSIISERFTVSPPKSFPGMAESTFLSRSFADQGV    180
VosA-gamma|348aa        LSVKIEGDFRLKFTLFEMRKEMVYYLKSIISERFTVSPPKSFPGMAESTFLSRSFADQGV    180
VosA-delta|140aa        LSVKIEGDFRLKFTLFEMRK----------------------------------------    140
VosA-epsilon|418aa      LSVKIEGDFRLKFTLFEMRKEMVYYLKSIISERFTVSPPKSFPGMAESTFLSRSFADQGV    168
VosA-zeta|338aa         LSVKIEGDFRLKFTLFEMRKEMVYYLKSIISERFTVSPPKSFPGMAESTFLSRSFADQGV    168
VosA-eta|336aa          LSVKIEGDFRLKFTLFEMRKEMVYYLKSIISERFTVSPPKSFPGMAESTFLSRSFADQGV    168
VosA-theta|128aa        LSVKIEGDFRLKFTLFEMRK----------------------------------------    128
                        ********************

                 pfam11754 Velvet |      |     cl21454 NADB_Rossmann superfamily
                        __________|      |___________________________________________

VosA-alpha|430aa        KLRIRKEPRTLIKRQVPRPEDYPQPLPPRSPDRTSMQMPGSAFGGYPAAGRDYGYYSNPV    240
VosA-beta|350aa         KLRIRKEPRTLIKRQVPRPEDYPQPLPPRSPDRTSMQMPGSAFGGYPAAGRDYGYYSNPV    240
VosA-gamma|348aa        KLRIRKEPRTLIKRQVPRPEDYPQPLPPRSPDRTSMQMPGSAFGGYPAAGRDYGYYSNPV    240
VosA-delta|140aa        ------------------------------------------------------------    140
VosA-epsilon|418aa      KLRIRKEPRTLIKRQVPRPEDYPQPLPPRSPDRTSMQMPGSAFGGYPAAGRDYGYYSNPV    228
VosA-zeta|338aa         KLRIRKEPRTLIKRQVPRPEDYPQPLPPRSPDRTSMQMPGSAFGGYPAAGRDYGYYSNPV    228
VosA-eta|336aa          KLRIRKEPRTLIKRQVPRPEDYPQPLPPRSPDRTSMQMPGSAFGGYPAAGRDYGYYSNPV    228
VosA-theta|128aa        ------------------------------------------------------------    128

                                 cl21454 NADB_Rossmann superfamily
                        ____________________________________________________________

VosA-alpha|430aa        KRQRTSMDFGPRGVYDADGRMRQVEAYPQAAAMYSNPPGAYQTPMVPGYPTGHAGVPDYS    300
VosA-beta|350aa         KRQRTSMDFGPRGVYDADGRMRQVEAYPQAAAMYSNPPGAYQTPMVPGYPTGHAGVPDYS    300
VosA-gamma|348aa        KRQRTSMDFGPRGVYDADGRMRQVEAYPQAAAMYSNPPGAYQTPMVPGYPTGHAGVPDYS    300
VosA-delta|140aa        ------------------------------------------------------------    140
VosA-epsilon|418aa      KRQRTSMDFGPRGVYDADGRMRQVEAYPQAAAMYSNPPGAYQTPMVPGYPTGHAGVPDYS    288
VosA-zeta|338aa         KRQRTSMDFGPRGVYDADGRMRQVEAYPQAAAMYSNPPGAYQTPMVPGYPTGHAGVPDYS    288
VosA-eta|336aa          KRQRTSMDFGPRGVYDADGRMRQVEAYPQAAAMYSNPPGAYQTPMVPGYPTGHAGVPDYS    288
VosA-theta|128aa        ------------------------------------------------------------    128

                 cl21454 NADB_Rossmann superfamily |
                        ___________________________|

VosA-alpha|430aa        LSYGMSAQVPQMQDPGAHGRSSQQATMQPFGMANAPGTPTADATGAMMQQGYPRGQYQTG    360
VosA-beta|350aa         LSYGMSAQVPQMQDPGAHGRSSQQATMQPFGMANAPGTPFWDRQQTRQER----------    350
VosA-gamma|348aa        LSYGMSAQVPQMQDPGAHGRSSQQATMQPFGMANAPGTPVCIDSHVSD------------    348
VosA-delta|140aa        ------------------------------------------------------------    140
VosA-epsilon|418aa      LSYGMSAQVPQMQDPGAHGRSSQQATMQPFGMANAPGTPTADATGAMMQQGYPRGQYQTG    348
VosA-zeta|338aa         LSYGMSAQVPQMQDPGAHGRSSQQATMQPFGMANAPGTPFWDRQQTRQER----------    338
VosA-eta|336aa          LSYGMSAQVPQMQDPGAHGRSSQQATMQPFGMANAPGTPVCIDSHVSD------------    336
VosA-theta|128aa        ------------------------------------------------------------    128
```

```
VosA-alpha|430aa        STILPPLQRSRNITQGSNGTPTRGYYDQSSQAATPILPSQPIGTNEAERYGLAPGQTAYE    420
VosA-beta|350aa         ------------------------------------------------------------    350
VosA-gamma|348aa        ------------------------------------------------------------    348
VosA-delta|140aa        ------------------------------------------------------------    140
VosA-epsilon|418aa      STILPPLQRSRNITQGSNGTPTRGYYDQSSQAATPILPSQPIGTNEAERYGLAPGQTAYE    408
VosA-zeta|338aa         ------------------------------------------------------------    338
VosA-eta|336aa          ------------------------------------------------------------    336
VosA-theta|128aa        ------------------------------------------------------------    128


VosA-alpha|430aa        HPGSADGTPR  430
VosA-beta|350aa         ----------  350
VosA-gamma|348aa        ----------  348
VosA-delta|140aa        ----------  140
VosA-epsilon|418aa      HPGSADGTPR  418
VosA-zeta|338aa         ----------  338
VosA-eta|336aa          ----------  336
VosA-theta|128aa        ----------  128
```
