# Supplementary material for: Transcriptomic Complexity of Aspergillus terreus Velvet Gene Family under the Influence of Butyrolactone I
Source: Microorganisms. 2017 Mar 14;5(1):12. doi: 10.3390/microorganisms5010012 (PMC5374389; doi:10.3390/microorganisms5010012)
Supplement: Supplementary file 1 [file microorganisms-05-00012-s001.zip › supplementary/Figure_S5.pdf]

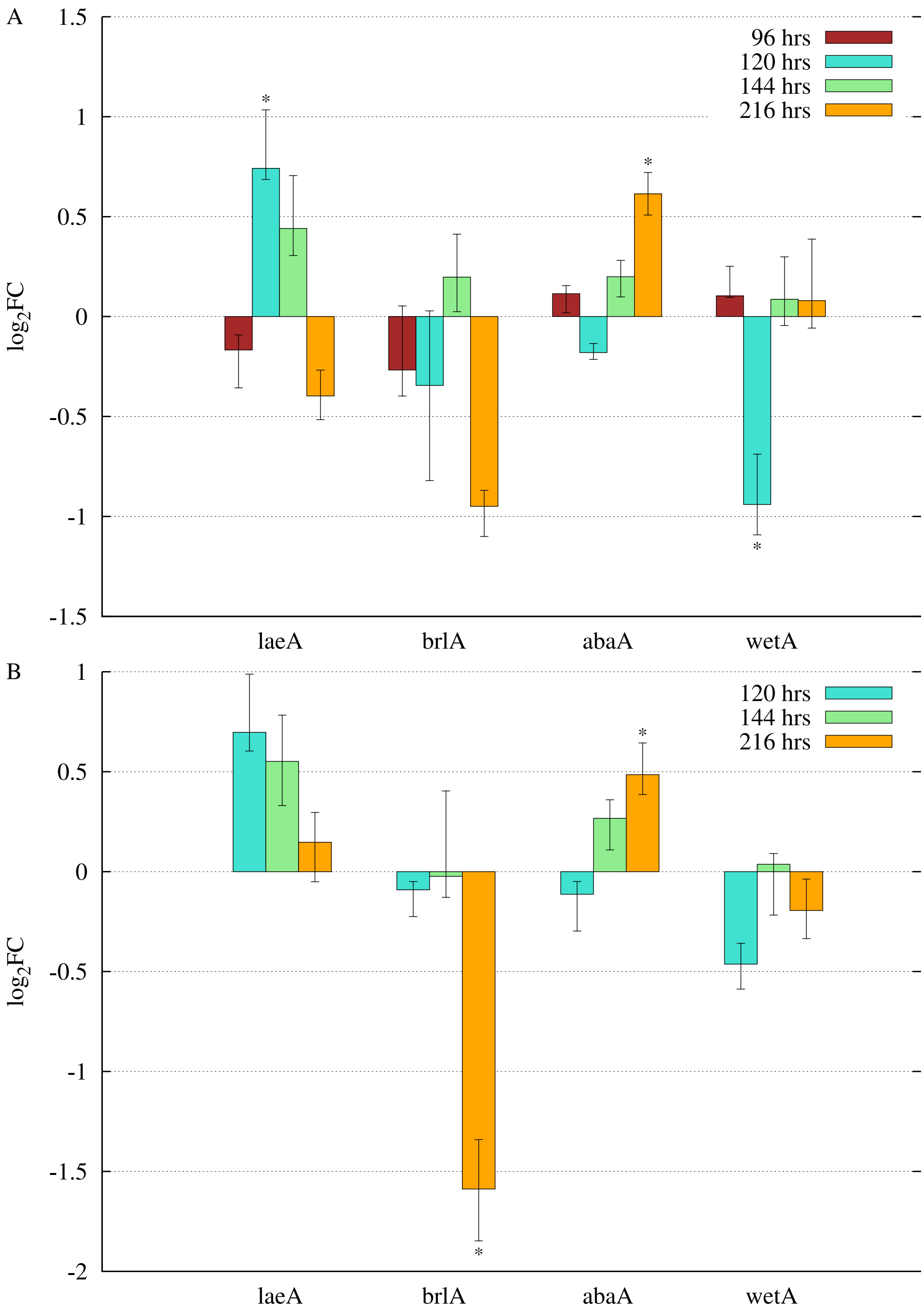

Figure S5. Butyrolactone I upregulates *A. terreus laeA* and appears to play a role in the transcription of the core conidiation regulators *brlA, abaA* and *wetA*. Exogenous butyrolactone I was added to the submerged culture of *A. terreus* MUCL38669 at (**A**) 96 hours and (**B**) 120 hours p.i. to the final concentration of 100 nM. Samples were analysed for gene expression using custom designed microarrays as described in the Methods. The bars represent the median values of the $\log_2$FC of treated samples (butyrolactone I was added) vs. control samples (no butyrolactone I added) and the error bars represent the maxima and minima of these $\log_2$FC values. **At least one of the four technical replicates indicates statistically significant differential expression in the three biological replicates adjusted P-value* ≤ 0.05 *and* $|\log_2 FC| \geq 0.5$.
