# Supplementary material for: Transcriptomic Complexity of Aspergillus terreus Velvet Gene Family under the Influence of Butyrolactone I
Source: Microorganisms. 2017 Mar 14;5(1):12. doi: 10.3390/microorganisms5010012 (PMC5374389; doi:10.3390/microorganisms5010012)
Supplement: Supplementary file 1 [file microorganisms-05-00012-s001.zip › supplementary/Figure_S6.pdf]

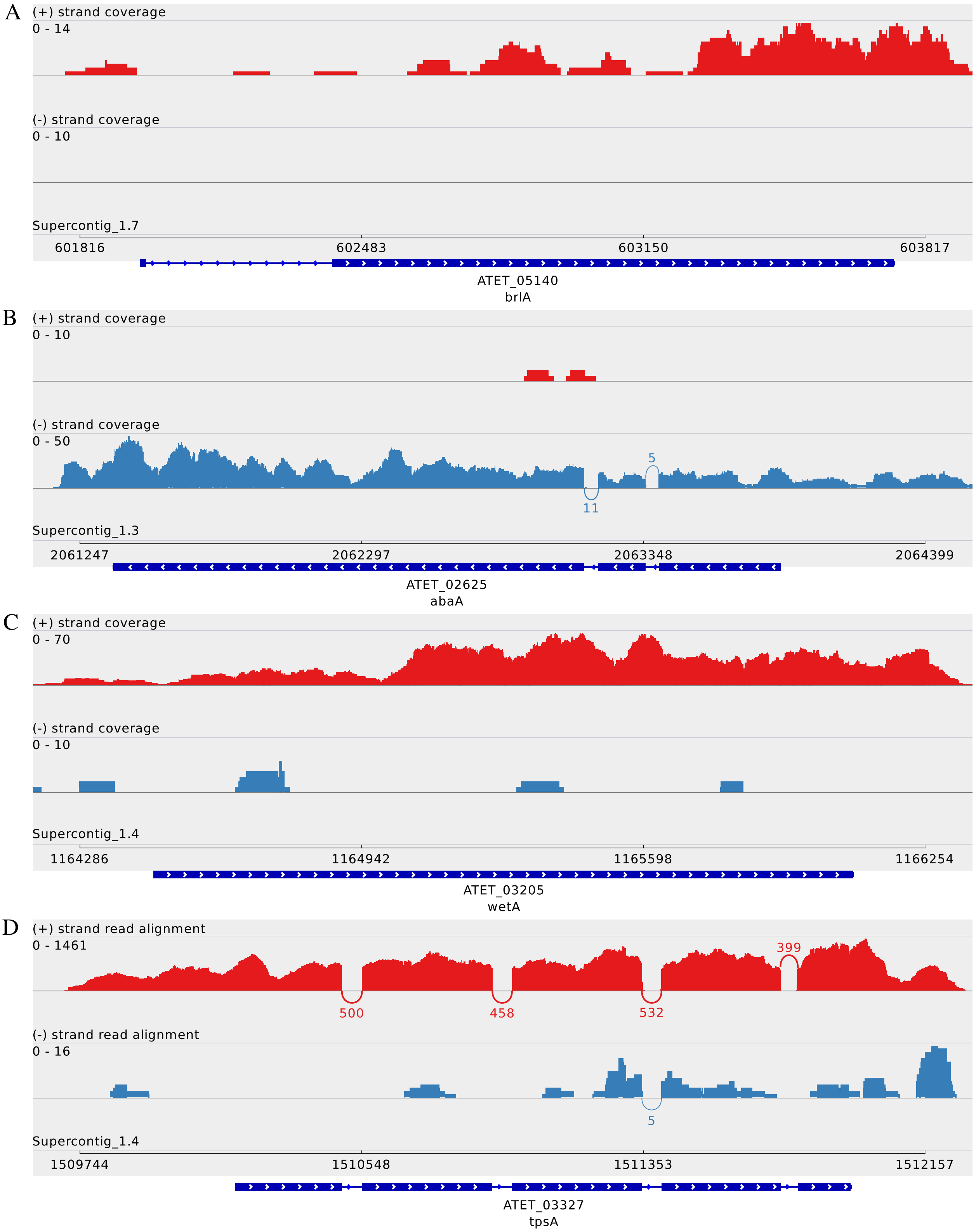

Figure S6. Sashimi plots of the transcription factors (**A**) *brlA* and (**B**) *abaA*, (**C**) the developmental regulatory protein *wetA* and (**D**) the alpha,alpha-trehalose-phosphate synthase 1, *tpsA* describing the strand-specific alignment coverage of the read sequences of *A. terreus* MUCL38669 over the corresponding genomic regions of NIH2624. The curved splice junctions represent the number of spliced reads indicating an intron at that specific location.
