# Supplementary material for: Transcriptomic Complexity of Aspergillus terreus Velvet Gene Family under the Influence of Butyrolactone I
Source: Microorganisms. 2017 Mar 14;5(1):12. doi: 10.3390/microorganisms5010012 (PMC5374389; doi:10.3390/microorganisms5010012)
Supplement: Supplementary file 1 [file microorganisms-05-00012-s001.zip › supplementary/File_S1.pdf]

Transcriptomic Complexity of *Aspergillus terreus* Velvet Gene Family under the Influence of Butyrolactone I

Elina K. Palonen[1], Sheetal Raina[2], Annika Brandt[1], Jussi Meriluoto[1], Tajalli Keshavarz[2] and Juhani Soini[3]

[1] Biochemistry, Faculty of Science and Engineering, Åbo Akademi University, Artillerigatan 6, FI-20520 Åbo, Finland
[2] School of Life Sciences, University of Westminster, London W1W 6UW, UK
[3] Faculty of Life Sciences and Business, Turku University of Applied Sciences, Lemminkäinengatan 30, FI-20520 Åbo, Finland

Supplementary file 1

Supplementary methods

Strain and chemicals

The *A. terreus* MUCL38669 was obtained from CABI Biosciences UK Centre, Surrey, United Kingdom. The materials used for culturing were obtained from Sigma-Aldrich Company Limited, Dorset, UK. The butyrolactone I was purchased from BIOMOL International, UK.

Culture conditions

The inoculation YME agar slants consisted of yeast extract 4 g/l, malt 10 g/l, glucose 4 g/l, agar 20 g/l and were incubated at 28°C for 7 days after which they were stored at 4°C. The developed spores were suspended in a sterile solution of 0.01 % Tween 80 (v/v). 10 ml of spore suspension (containing 10^7 spores/ml) was used to inoculate 100 ml of inoculation medium. Inoculation medium consisted of corn steep liquor 5 g/l, tomato paste 40 g/l, oat flour 10 g/l, dextrose 10 g/l, trace element solution 10 ml/l with pH 6.8. The element solution contained $FeSO_4 \cdot 7H_2O$ 1 g/l, $MnSO_4 \cdot 4H_2O$ 1 g/l, $CuCl_2 \cdot 2H_2O$ 0.025 g/l, $CaCl_2 \cdot H_2O$ 0.1 g/l, H_3BO_3 0.056 g/l, $(NH_4)_6Mo_7O_{24} \cdot 4H_2O$ 0.019 g/l, $ZnSO_4 \cdot 7H_2O$ 0.2 g/l. The inoculation medium was shaken at 220 rpm and 27°C for 25 hours in a 250 ml Erlenmayer flask. 10 ml of this inoculation medium was used to inoculate 100 ml of GPY-L production medium and was shaken at 220 rpm and 27°C for 216 h in a 250 ml Erlenmayer flask. The GPY-L medium consisted of glucose 25 g/l, peptonised milk 24 g/l, yeast extract 2.5 g/l, lactose 50 g/l.

Gene expression analysis using microarrays

The microarray slides that had been used were of custom Agilent 4 x 44K format (Agilent Technologies Inc., Wilmington, DE, USA) for which the total RNA of MUCL38669 had been extracted using Qiagen RNeasy Plant Mini Kit (Qiagen, Sollentuna, Sweden) with a modified protocol as described in our previous study [1] and labelled as instructed in Agilent One-Color Microarray-based Gene Expression Analysis Protocol, version 5.5. Two separate oligonucleotide probes of 60 nt in length per one available transcript of *A. terreus* NIH2624 had been designed and constructed in two technical replicates by eArray of Agilent Technologies. The extracted one-color signal intensities of the microarray images that had been obtained in our previous study were further analysed using the statistical computing language and environment R (R Development Core Team (2011)) [2] version 2.14.1 and the linear models for microarray data (limma) package [3, 4]. The data background was corrected with "normexp" and "saddle" method after which the data was normalised between arrays with "quantile" algorithm and $\log_2$ transformed [5]. Probes that had been determined as not found by the Feature Extraction Software (version 9.5.3.1, Agilent Technologies Inc., Wilmington, DE, USA) were removed from the data. Next, the already designed probes were bioinformatically filtered in order to exclude the unreliable ones from the further analysis as described in the main Methods section. The following statistical gene expression analysis included only the normalised,

filtered data and was performed exactly as in our previous study [1]. Shortly, a moderated t-statistic was applied, followed by empirical Bayesian statistics with Benjamini-Hochberg's method to adjust the p-values for multiple testing after fitting a linear model to each gene by applying the limma package [6].

Evaluation of the normalised microarray data reliability after the microarray probe filtering

One of the aims of this study is to improve the reliability of the previous microarray gene expression results obtained under the influence of exogenously added butyrolactone I [1]. The microarray probe design of that study was based on the gene annotation of strain NIH2624, whereas the strain used in the cultures was MUCL38669. In this study, the reliability of these microarray results was verified by aligning these previously designed probe sequences with the in this study sequenced transcriptome in order to extract specifically the probes that fulfill the specified alignment thresholds (see the main Methods section for further filtering details). The purpose of the filtering was to focus the microarray gene expression analysis to the thereby verified probes, thus allowing the reliable gene expression results to be examined. As a result, an average 65 % of the sequenced, expressed gene transcripts have a reliable probe available in this study. Specifically, the filtering process equalised the lower quartiles of the normalised sample specific gene expression values as well as relocated the gene expression medians on a higher and more centered expression level within the lower and higher quartiles. In addition, the gene expression medians of all the samples shared same level ensuring the comparability of the samples' gene expression values (Fig. S2 and S3).

Strand-specific transcriptome sequencing

The total RNA was quality checked using Agilent 2100 Bioanalyzer, Eukaryote Total RNA Pico assay (Agilent Technologies Inc., Waldbronn, Germany) and a ND-1000 NanoDrop Spectrophotometer (NanoDrop Technologies Inc., DE, USA) prior to mRNA purification. Total RNA was purified to obtain mRNA using Dynabeads mRNA Purification Kit (Cat. No. 610.06, Invitrogen Dynal AS, Oslo, Norway) and a DynaMag-2 magnet stand (Cat. No. 123.21D, Invitrogen Dynal AS, Oslo, Norway) with the following modified protocol. 64 µg of total RNA was diluted with milli-Q water to 100 µl and was heated at 65℃ for 5 minutes after which it was chilled on ice. Next, 200 µl of Dynal oligo(dT) beads (Cat. No. 610-06) was washed twice with 100 µl of Binding Buffer (20 mM Tris-HCl pH 7.5, 1.0 M LiCl and 2 mM EDTA) by removing the supernatant each time. The Dynal beads were resuspended in 100 µl of Binding Buffer where 100 µl of the heated and chilled total RNA was added followed by rotation at room temperature for 5 minutes after which the supernatant was removed. The Dynal beads with bound polyA mRNA were washed twice with 200 µl of Washing Buffer B (10 mM Tris-HCl pH 7.5, 0.15 M LiCl, 1 mM EDTA). Next, the supernatant was removed and 20 µl of 10 mM Tris-HCl was added and the beads were heated at 80℃ for 2 minutes to elute mRNA after which the beads were immediately put on the magnet stand and the supernatant that contained the eluted mRNA was transferred to a new tube containing 80 µl of 10 mM Tris-HCl. 100 µl of Binding Buffer was added to the remaining beads. Next, the eluted

mRNA was subjected to a second purification round, where the eluted mRNA was heated at 65°C for 5 minutes and chilled on ice after that. The remaining beads after mRNA elution were washed twice with 100 µl of Binding Buffer, and 100 µl of Binding Buffer was added to the beads after the wash. The eluted mRNA (100 µl) was added to the washed beads in 100 µl Binding Buffer and was rotated for 5 minutes in room temperature. Next, the supernatant was removed and the beads were washed twice with 200 µl of Washing Buffer B. The supernatant was removed and 12 µl of 10 mM Tris-HCl was added to the beads, and heated at 80°C for 2 minutes to elute the mRNA. Immediately after the heating, the beads were put on the magnet stand and the supernatant containing the eluted mRNA was transferred to a new 200 µl PCR tube to be fragmented. The quality of the mRNA was checked with ND-1000 NanoDrop Spectrophotometer and Agilent Bioanalyzer using mRNA Pico Assay Kit. Next, the mRNA was fragmented using Ambion RNA Fragmentation Reagents following the manufacturer's instructions for 10 µl reaction volume except for the incubation time was shortened to 2.5 min (Cat. No. AM8740, Ambion Inc.). The fragmented mRNA was purified using Bio-Rad Micro Bio-Spin 30 Columns, RNase-free in Tris Buffer kit following the manufacturer's instructions (Cat. No. 732-6250, Bio-Rad Laboratories Inc., CA, USA.) followed by quality checking using ND-1000 NanoDrop Spectrophotometer and mRNA Pico Assay Kit for Agilent Bioanalyzer. The first strand of cDNA was synthesised from the fragmented mRNA using Invitrogen reagents (Invitrogen Co., CA, USA) as follows: 1 µl of Random Hexamer Primers (3 µg/µl, Cat. No. 48190-011), 190 ng of fragmented mRNA, 4 µl of 5x first strand buffer (Cat. No. 18064-014), 2 µl of DTT (100 mM, Cat. No. 18064-014), 1 µl of dNTP Mix (10 mM, Cat. No. 18427-013) were added into a 200 µl thin wall PCR tube and mixed. Next, the mixture was prepared for reverse trascriptase addition by the following thermocycler (Bio-Rad S1000 Thermal Cycler, Bio-Rad Laboratories Inc.) program: 1 minute at 98°C, 5 minutes at 70°C to denaturate RNA, followed by decreasing the temperature down to 15°C with 0.1°C steps and incubating at 15°C for 30 minutes to allow random primer annealing and was held at 15°C until the reverse transcriptase was loaded. Next, 1 µl of RNaseOUT (40 U/µl, Cat. No. 10777-019), 1 µl of actinomycin D (120 ng/µl, Cat. No. A1410-2MG, Sigma-Aldrich Co., MO, USA) and 1 µl of SuperScript III Reverse Transcriptase (200 U/µl, Cat. No. 18080-093) were added and mixed with the pipet. The first strand cDNA was synthesised with the following thermocycler program: increasement of the temperature up to 25°C with 0.1°C steps followed by incubation of 10 minutes at 25°C, followed by temperature increasement up to 42°C with 0.1°C steps and incubation at 42°C for 45 minutes. This was followed by temperature increasement up to 50°C and incubation for 25 minutes at 50°C. Next, to inactivate the reverse transcriptase the temperature was increased up to 75°C and incubated for 15 minutes after which it was held at 15°C before putting the tube on ice. The synthesised first strand cDNA was purified and the dNTPs removed using Bio-Rad Micro Bio-Spin 30 Columns, RNase-free in Tris Buffer kit following the manufacturer's instructions. The quality of the first strand cDNA was checked using ND-1000 NanoDrop Spectrophotometer and Agilent Bioanalyzer mRNA Pico Assay Kit. In order to preserve the strand-specificity during the second strand cDNA synthesis dUTPs were used instead of dTTPs. First, 66.5 µl of milli-Q water was added to the first strand cDNA eluate, followed by preparing the second strand synthesis buffer as follows: 4 µl of 5x first strand buffer (Cat. No. 18064-014,

Invitrogen Co.), 2 µl of DTT (100 mM, Cat. No. 18064-014, Invitrogen Co.), 30 µl of 5x second strand reaction buffer (Cat. No. 10812-014, Invitrogen Co.), 4 µl of dATP, dCTP, dGTP each (10 mM, Cat. No. N808-0007, Applied Biosystems of Roche Molecular Systems Inc., NJ, USA) and 2 µl of dUTP (20 mM, Cat. No. N808-0068, Applied Biosystems of Roche Molecular Systems Inc.) were mixed and added to the cDNA eluate. It was mixed and incubated on ice until it was chilled. Next, following enzymes (New England BioLabs Inc., MA, USA) were added: 1 µl of E. coli DNA Ligase (10 U/µl, Cat. No. M0205S), 4 µl of E. coli DNA Polymerase I (10 U/µl, Cat. No. M0209S), 0.5 µl of E. coli RNase H (5 U/µl, Cat. No. M0297S) and mixed gently. The mixture was incubated for 2 hours at 16℃ in the Bio-Rad thermocycler. The obtained ds cDNA was purified using Qiagen QIAquick PCR Purification Kit (Qiagen, Sollentuna, Sweden, Cat. No. 28104) following the manufacturer's instructions with 10 µl addition of 3 M Sodium Acetate before the sample was transferred to the QIAquick spin column and eluted in 50 µl of EB buffer. The quality of the purified ds cDNA was checked with ND-1000 NanoDrop Spectrophotometer and Agilent Bioanalyzer High Sensitivity DNA Assay. The obtained strand-specific ds cDNA (220 ng) was processed to sequencing according to the Illumina sample preparation instructions (Preparing Samples for Sequencing of mRNA) with an additional digestion with 1 Unit of Uracil-N-Glycosylase (Cat. No. N808-0096, Applied Biosystems of Roche Molecular Systems Inc.) for 15 minutes at 37℃ before the library amplification step in the Illumina instructions.

Quality evaluation of the transcriptome sequence raw data

The obtained paired-end sequence reads were trimmed using a FASTX-Toolkit (version 0.0.13) to improve the read quality by removing the known multiplex adapters, PCR primers and low quality parts [7]. The RNA-seq raw data quality was estimated before and after the trimming using FastQC programme (version 0.10.1) [8] prior to the transcriptome assembly. The sequence quality describing characteristics included mean GC base proportions, per base Phred scores, read duplication approximations, sequence overrepresentation and sequence kmer enrichment evaluation. The resulting 55 % GC content describes approximately equal base amounts amongst the sequence reads, Phred score medians being > 35 (based on Illumina 1.5 encoding fastq format raw data) indicate that approximately > 99.97 % of the sequenced bases are likely to be accurate. Although the occurrence of read duplication is high even after the trimming procedure (> 65 %) and the amounts of enriched 5 bp long sequence kmers are high in both paired-end raw data as well, no significant amount of overrepresented read sequences were observed (i.e. the number of duplicate reads remained under 0.1 % of the total number of reads) (Table S1). These apparently contradicting raw data observations were clarified by analysing the distribution of the normalised accumulation numbers of the assembled transcripts of the pooled RNA samples (represented as pooled FPKM values) using the "quantile" function of R software [2]. The distribution of the obtained pooled FPKM values over the quantiles of the number of the sequenced genes reveals the highest accumulation (FPKM medians 206, 461 and 32141) to occur for few quantile fractions (0.95, 0.975 and 1, respectively), indicating the enrichment of these transcripts. The highest pooled FPKM value comprises 5 % of the total number of accumulated transcripts (FPKM of 63388) confirming the enrichment of only few specific

transcript(s) (File S2 and Fig. S1). The significant majority of these obtained normalised transcript amounts of the pooled samples are within an accumulation range of $0 - 500$ FPKM (Fig. S1). The high sequence duplication level as indicated by FastQC analysis may be explained by the applied procedure of the programme, where the analysed reads of 101 bp in length are truncated to 50 bp in order to reduce the computing conditions, which increases the possibility of apparent occurrence of read duplication.

References

[1] Raina, S., de Vizio, D., Palonen, E.K., Odell, M., Brandt, A.M., Soini, J.T., Keshavarz, T.: Is quorum sensing involved in lovastatin production in the filamentous fungus *Aspergillus terreus*? Process Biochem **47**(5), 843–852 (2012)

[2] R: A language and environment for statistical computing, Vienna, Austria. Accessed 2012 (2011). http://www.r-project.org/

[3] Smyth, G.K.: limma: linear models for microarray. In: Gentleman, R., Carey, V.J., Huber, W., Irizarry, R.A., Dudoit, S. (eds.) Bioinformatics and Computational Biology Solutions Using R and Bioconductor. Statistics for Biology and Health, pp. 397–420. Springer, New York, NY (2005)

[4] Smyth, G.K.: Linear models and empirical bayes methods for assessing differential expression in microarray experiments. Stat Appl Genet Mol Biol **3**(1), 1–25 (2004)

[5] Smyth, G.K., Speed, T.: Normalization of cDNA microarray data. Methods **31**(4), 265–273 (2003)

[6] Benjamini, Y., Hochberg, Y.: Controlling the false discovery rate: a practical and powerful approach to multiple testing. J Roy Stat Soc B Met **57**(1), 289–300 (1995)

[7] Hannon, G.J.: FASTX toolkit. Version 0.0.13. http://hannonlab.cshl.edu/

[8] Simon, A.: FastQC: a quality control tool for high throughput sequence data. Version 0.10.1. http://www.bioinformatics.babraham.ac.uk/projects/fastqc/
