# Supplementary material for: Transcriptomic Complexity of Aspergillus terreus Velvet Gene Family under the Influence of Butyrolactone I
Source: Microorganisms. 2017 Mar 14;5(1):12. doi: 10.3390/microorganisms5010012 (PMC5374389; doi:10.3390/microorganisms5010012)
Supplement: Supplementary file 1 [file microorganisms-05-00012-s001.zip › supplementary/Tables_S1-S2.pdf]

Table S1. Information of the sequence quality [a].

Paired end reads (PE)	Number of reads	Median read length (bp)	GC %	Per base sequence qualities: Phred score median (lower quartile)	Read duplication level [b]	Number of over-represented reads [c]	Number of enriched 5 bp kmers [b]
raw PE 1	18752589	101	55	≥ 35.0 (≥ 30.5)	≥ 78.26 %	0	62
raw PE 2	18752589	101	55	≥ 33.5 (≥ 27.5)	≥ 80.07 %	0	59
trimmed PE 1	18204865	101	55	≥ 36.5 (≥ 33.5)	≥ 65.68 %	0	32
trimmed PE 2	18204865	101	55	≥ 35.2 (≥ 32.2)	≥ 79.99 %	0	60

[a] *FastQC programme (version 0.10.1) was used to obtain the values; see File S1 for details*

[b] *Few specific transcripts were enriched as revealed by the distribution of the transcript FPKM values (Fig. S1)*

[c] *A read is overrepresented if the number of its duplicates > 0.1 % of total number of read sequences*

Table S2. Facts of the assembled transcriptome.

Total number of transcripts	41695
Total length of the assembly [a]	41.92 Mbp
Transcript minimum length	125 bp
Transcript maximum length	13056 bp
Transcript N50	1796 bp
Approximate sequence coverage [b]	88

[a] *The assembly contains also putative splice variants and antisense sequences*

[b] *Total number of trimmed reads (36.4 Million) with median length of 101 bp / assembly length (Mbp)*
