# Supplementary material for: Transcriptomic Complexity of Aspergillus terreus Velvet Gene Family under the Influence of Butyrolactone I
Source: Microorganisms. 2017 Mar 14;5(1):12. doi: 10.3390/microorganisms5010012 (PMC5374389; doi:10.3390/microorganisms5010012)
Supplement: Supplementary file 1 [file microorganisms-05-00012-s001.zip › supplementary/Table_S3.pdf]

Table S3. ORF and intron locations of *A. terreus* MUCL38669 velvet family members and *laeA* transcripts on the strain NIH2624 genome.

Gene	ORF location	Upstream intron	Intron locations	Genomic contig of NIH2624
veA	1243137-1244906(-)	ND	-	Supercontig_1.1
velB	1816607-1817856(-)	yes	1816881-1816935(-)	Supercontig_1.6
			1817419-1817477(-)	
			1817558-1817610(-)	
			1818127-1818392(-) [a]	
velC	2136789-2138241(+)	yes	2136283-2136443(+) [a]	Supercontig_1.1
			2137205-2137331(+)	
		no	2137205-2137331(+)	
vosA-α	1207293-1209313(+)	no	1207343-1207443(+)	Supercontig_1.5
			1207512-1207615(+)	
			1207686-1207763(+)	
			1207910-1207975(+)	
			1208061-1208149(+)	
			1208197-1208254(+)	
			1208364-1208414(+)	
			1208740-1208861(+)	
			1208979-1209037(+)	
vosA-β	1207293-1209062(+)	no	1207343-1207443(+)	
			1207512-1207615(+)	
			1207686-1207763(+)	
			1207910-1207975(+)	
			1208061-1208149(+)	
			1208197-1208254(+)	
			1208364-1208414(+)	
			1208740-1208861(+)	
			1208979-1209026(+)	
vosA-γ	1207293-1209008(+)	no	1207343-1207443(+)	
			1207512-1207615(+)	
			1207686-1207763(+)	
			1207910-1207975(+)	
			1208061-1208149(+)	
			1208197-1208254(+)	
			1208364-1208414(+)	
			1208740-1208861(+)	
vosA-δ	1207293- 1208064(+)	no	1207343-1207443(+)	
			1207512-1207615(+)	
			1207686-1207763(+)	
			1207910-1207975(+)	

Gene	ORF location	Upstream intron	Intron locations	Genomic contig of NIH2624
vosA-ε	1207430-1209313(+)	yes	1207343-1207406(+) [a]	Supercontig_1.5
			1207512-1207615(+)	
			1207686-1207763(+)	
			1207910-1207975(+)	
			1208061-1208149(+)	
			1208197-1208254(+)	
			1208364-1208414(+)	
			1208740-1208861(+)	
			1208979-1209037(+)	
vosA-ζ	1207430-1209062(+)	yes	1207343-1207406(+) [a]	
			1207512-1207615(+)	
			1207686-1207763(+)	
			1207910-1207975(+)	
			1208061-1208149(+)	
			1208197-1208254(+)	
			1208364-1208414(+)	
			1208740-1208861(+)	
			1208979-1209026(+)	
vosA-η	1207430-1209008(+)	yes	1207343-1207406(+) [a]	
			1207512-1207615(+)	
			1207686-1207763(+)	
			1207910-1207975(+)	
			1208061-1208149(+)	
			1208197-1208254(+)	
			1208364-1208414(+)	
			1208740-1208861(+)	
vosA-θ	1207430-1208064(+)	yes	1207343-1207406(+) [a]	
			1207512-1207615(+)	
			1207686-1207763(+)	
laeA-α, variant 1	1900810-1902006(+)	yes	1900017-1900079(+) [a]	Supercontig_1.1
			1901025-1901116(+)	
laeA-α, variant 2		no	1901025-1901116(+)	
laeA-β, variant 1	1901184-1902006(+)	yes	1900017-1900079(+) [a]	
laeA-β, variant 2		no	-	

[a] *upstream intron*

ND; not detected
