# Supplementary material for: Transcriptomic Complexity of Aspergillus terreus Velvet Gene Family under the Influence of Butyrolactone I
Source: Microorganisms. 2017 Mar 14;5(1):12. doi: 10.3390/microorganisms5010012 (PMC5374389; doi:10.3390/microorganisms5010012)
Supplement: Supplementary file 1 [file microorganisms-05-00012-s001.zip › supplementary/Table_S4.pdf]

Table S4. Sequenced transcript details regarding the core regulators of conidiation (*brlA, abaA, wetA*) and spore viability providing trehalose biosynthase (*tpsA*).

Gene	ORF length (bp)	Gene length (bp) [a]	Transcript length (bp) [b]	Number of exons (introns)	Residue identity (%) with *A. nidulans* ortholog [c]
brlA	NA	NA	NA	NA	75 [d]
abaA	2391	2491	5745 [e]	3 (2)	62
wetA	1632	1632	2700	-	61
tpsA	1542	1759	2435	5 (4)	90

[a] *based on the genomic region of strain NIH2624 between 5' and 3' of the corresponding gene*

[b] *derived from the corresponding sequenced transcript of strain MUCL 38669, including sequenced up- and downstream regions with upstream introns if present*

[c] *obtained by BLASTP analysis*

[d] *the ID % is based on brlA annotation of strain NIH2624*

[e] *sequenced transcript covers also ATEG_02626 gene of NIH2624 annotation*
NA; not available for strain MUCL 38669
