# Supplementary figures and images for: Transcriptomic Complexity of Aspergillus terreus Velvet Gene Family under the Influence of Butyrolactone I
Source: Microorganisms. 2017 Mar 14;5(1):12. doi: 10.3390/microorganisms5010012 (PMC5374389; doi:10.3390/microorganisms5010012)

**Figure S2. Gene expression data in quartiles after normalisation**

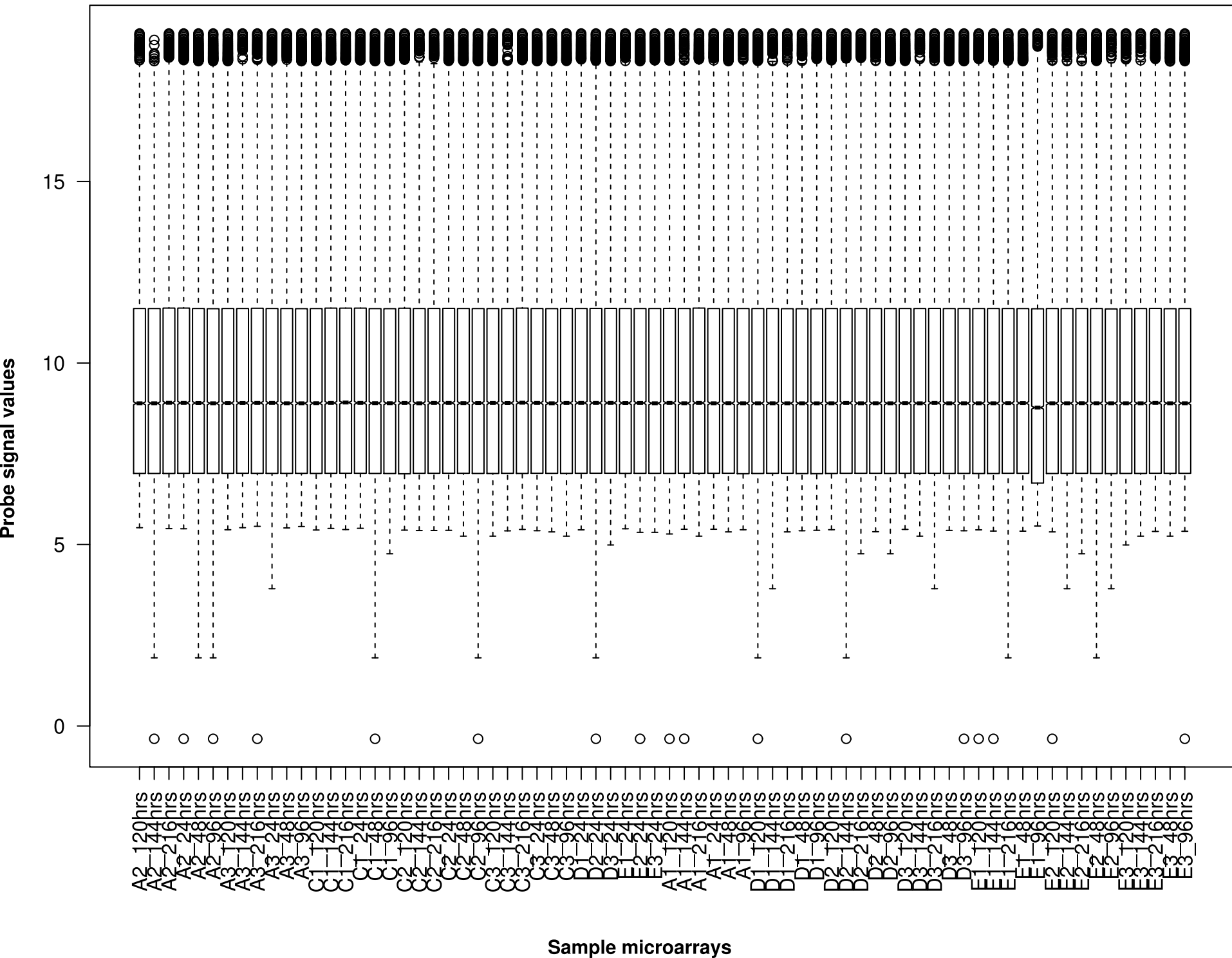

Supplement: Supplementary file 1 [file microorganisms-05-00012-s001.zip › supplementary/Figure_S2.pdf]
